# Hepatocellular carcinoma-derived high mobility group box 1 triggers M2 macrophage polarization via a TLR2/NOX2/autophagy axis

**DOI:** 10.1038/s41598-020-70137-4

**Published:** 2020-08-12

**Authors:** Dong-Jer Shiau, Wan-Ting Kuo, Goutham Venkata Naga Davuluri, Chi-Chang Shieh, Pei-Jane Tsai, Chien-Chin Chen, Yee-Shin Lin, Yi-Zhen Wu, Yu-Peng Hsiao, Chih-Peng Chang

**Affiliations:** 1 Department of Microbiology and Immunology, College of Medicine, National Cheng Kung University, Tainan, 701 Taiwan; 2 The Institute of Basic Medical Sciences, College of Medicine, National Cheng Kung University, Tainan, 701 Taiwan; 3 Institute of Clinical Medicine, National Cheng Kung University Hospital, Tainan, 701 Taiwan; 4 Division of Allergy, Immunology and Rheumatology, Department of Pediatrics, College of Medicine, National Cheng Kung University Hospital, Tainan, 701 Taiwan; 5 Department of Medical Laboratory Science and Biotechnology, National Cheng Kung University, Tainan, 701 Taiwan; 6 Department of Pathology, Chia-Yi Christian Hospital, Chiayi, 600 Taiwan; 7 Department of Cosmetic Science, Chia Nan University of Pharmacy and Science, Tainan, 701 Taiwan; 8 Center of Infectious Disease and Signaling Research, National Cheng Kung University, Tainan, 701 Taiwan

**Keywords:** Cancer, Cancer microenvironment, Gastrointestinal cancer, Tumour immunology, Immunology, Innate immune cells, Tumour immunology

## Abstract

In many human cancers, including hepatocellular carcinoma (HCC), high density of infiltrating tumor-associated macrophages (TAM) is associated with poor prognosis. Most TAMs express a M2 phenotype subsequently supporting tumor growth. How tumor cells polarize these TAMs to a pro-tumor M2 phenotype is still poorly understood. Our previous studies have revealed that a Toll-like receptor 2 (TLR2)-dependent autophagy triggered by hepatoma-derived factors down-regulates NF-κB p65 and drives M2 macrophage differentiation. However, the underlying mechanisms and potential hepatoma-derived TLR2 ligands are not clear. Here, we provide evidence to reveal that NADPH oxidase 2 (NOX2)-dependent reactive oxygen species (ROS) generation is crucial for HCC-induced autophagy, NF-κB p65 down-regulation and M2 phenotype polarization in primary macrophages. This NOX2-generated ROS production in abolished in TLR2-deficient macrophages. HCC-derived or recombinant high-mobility group box 1 (HMGB1) is able to trigger this TLR2-mediated M2 macrophage polarization. Blockage of HMGB1 and ROS by inhibitors, ethyl pyruvate and N-acetylcysteine amide, respectively, significantly reduces both M2 macrophage accumulation and liver nodule formation in HCC-bearing mice. Our findings uncover a HMGB1/TLR2/NOX2/autophagy axis to trigger M2 macrophage polarization in HCC that can be considered as a novel therapeutic target for treating HCC.

## Introduction

Leukocyte subpopulations have been found to accumulate within tumors. One prominent population is tumor-associated macrophages (TAM), and although immunologists think that the presence of TAM is evidence of a host immune response against growing tumors, many studies have demonstrated that these TAMs promote tumor progression^1^. Accordingly, in human cancers, such as hepatocellular carcinoma (HCC), a high density of infiltrating TAM is associated with poor prognosis^2^. These observations raise the possibility of targeting TAM in a therapeutic strategy for tumor treatment.

TAM has been classified into classic (M1) and alternative (M2) macrophages, according to their gene and protein profiling. M1 macrophages typically express proinflammatory cytokines (e.g. IFN-β, IL-12, TNF, IL-6, and IL-1β) and MHC class II molecules, and carry a therapeutic benefit, promoting anti-tumor activities^3^. On the other hand, M2 phenotype macrophages show IL-10^high^, IL-12^low^, arginase-1^high^, mannose receptor (CD206)^high^, scavenger receptors (CD204)^high^, MHC-II^low^, inducing an immunosuppressive tumor environment and hence promoting tumor progression^4^. It has been suggested that tumor cells can secrete chemotactic factors to actively attract circulating monocytes to tumor sites. Those recruited monocytes will differentiate into immunosuppressive TAM in tumor-microenvironments. Nevertheless, how macrophages differentiate to M2-type TAM in tumor microenvironments is not fully understood. In our previous study, we demonstrated that hepatoma cell-derived factors are able to trigger M2 macrophage polarization via toll-like receptor (TLR) 2 signaling. This TLR2 signaling pathway was found to down-regulate NF-κB p65, a M1-promoting transcription factor, via p62-mediated selective autophagy^5^. However, the underlying mechanisms and potential hepatoma-derived TLR2 ligands still need to be clarified.

NADPH oxidase (NOX) is a membrane-bound enzyme and is composed of five subunit proteins, including gp91^phox^, p22^phox^ p47^phox^, p67^phox^, and Rac. It is widely expressed in phagocytes (also known as NOX2), generating reactive oxygen species (ROS) which act against invading pathogens by regulating inflammasome, autophagy and type-1 interferon signaling^6^. In addition to defending against pathogens, NOX has been shown to be involved in tumor-associated immune cells polarization and differentiation, including myeloid-derived suppressor cells and TAM^7–9^. NOX-generated ROS or NOX itself has been reported to regulate M2 macrophage polarization after IL-4 and M-CSF stimulation. Blockage of NOX-generated ROS or NOX deficiency inhibits M2 macrophage polarization and lung cancer growth in a mouse model^8,9^. However, the contribution of NOX-derived ROS in hepatoma-induced M2 macrophage polarization remains unclear.

High-mobility group box 1 (HMGB1) is a member of the high-mobility group protein family and was originally characterized as a non-histone, nuclear DNA-binding protein^10^. However, HMGB1 is able to translocate to cytosol and hence secrete from the cells in response to various kinds of stimulation. Secreted HMGB1 will be further recognized by several damage-associated molecular patterns (DAMP) receptors, including TLR2, TLR4, TLR9, and the receptor for advanced glycation end-products (RAGE) to trigger inflammation, tissue repair and macrophage polarization^11,12^. High levels of HMGB1 in HCC patients are found to correlate with disease severity^13^.

In this study we show that hepatoma-derived HMGB1 stimulates ROS via the TLR2/NOX2 axis to trigger autophagy-regulated M2 macrophage polarization. Blockage of ROS and HMGB1 reduces accumulation of M2 macrophages within the hepatoma and attenuates hepatoma growth in mice. These results suggest that hepatoma-derived HMGB1 is able to regulate TAM polarization and may serve as a therapeutic target for hepatoma treatment.

## Results

### NOX2-dependent ROS generation is responsible for autophagy induction and M2 macrophage polarization

**Figure 1:**
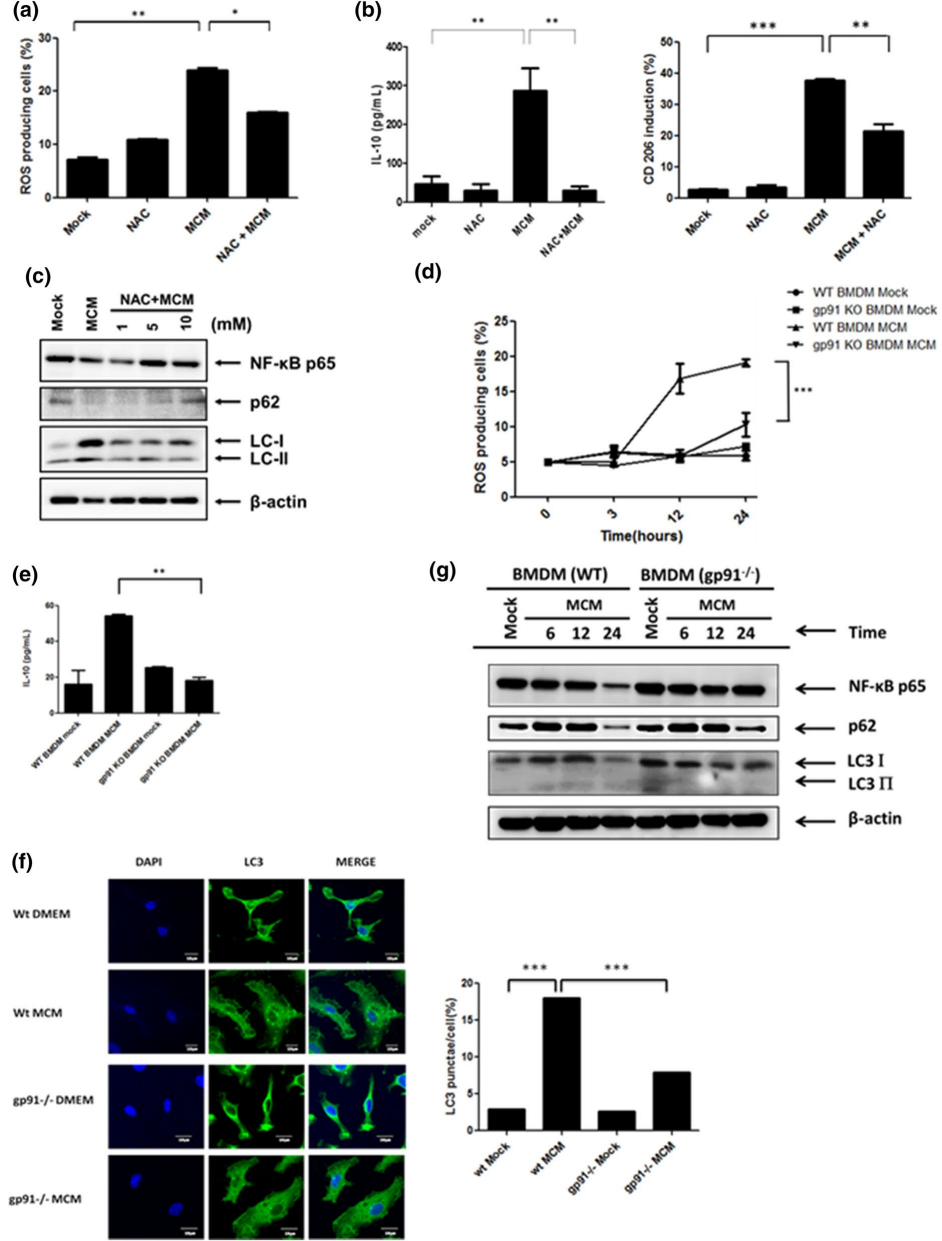
NOX2-generated ROS is responsible for hepatoma-induced autophagy and M2 macrophage polarization. (**a**) BMDMs were pretreated with or without N-acetyl cysteine (NAC, 10 mM) for 30 min and then incubated with MCM for another 24 h. The ROS induction was analyzed by flow cytometry. (**b**) BMDMs were pretreated with or without N-acetyl cysteine (NAC, 10 mM) for 30 min and then incubated with MCM for another 24 h. Supernatants and cell lysates were then collected for determining IL-10 expression by ELISA and CD206 expression by flow cytometry, respectively. (**c**) BMDMs were treated with MCM in the presence or absence of NAC (1, 5, or 10 mM) for 24 h. Cell lysates were collected to determine the expression of NF-*κ*B p65, LC3-I/II, and p62 by Western blotting. (**d**) Wild type (WT) or gp91^−/−^ BMDMs were treated with MCM for the indicated time and the ROS production was determined by flow cytometry. (**e**) WT or gp91^−/−^ BMDMs were treated with MCM for 24 h and supernatants were collected to analyze the production of IL-10 by ELISA. (**f**) NOX2-depedent autophagy is induced by MCM. WT or gp91^−/−^ BMDMs were treated with MCM for 12 h and the LC3 punctation was observed and quantified by confocal microscopy. (**g**) WT or gp91^−/−^ BMDMs were treated with MCM for the indicated time. Their cell lysates were then collected to determine the expression of NF-κB p65, LC3-I/II, and p62 by Western blotting. *p < 0.05; **p < 0.001; ***p < 0.0001.

We have previously reported that hepatoma-derived factors from a mouse hepatoma cell line ML-1_4a_ trigger autophagy-mediated M2 macrophage polarization^5^. Here, we went further to study the mechanisms in regulating this autophagy mediated M2 macrophage polarization. It has been shown that the ROS is involved in autophagy formation and plays an important role in IL-4/M-CSF-triggered M2 polarization^8^. Hence, we tried to investigate the role of ROS in hepatoma cell ML-1_4a_ conditioned medium (MCM)-triggered M2 polarization. From the data obtained here, we were able to detect ROS generation in MCM-treated BMDMs (Fig. 1a). Inhibition of ROS by a scavenger, N-acetylcysteine amide (NAC), decreased MCM-induced IL-10 production and CD206 up-regulation (Fig. 1b). In addition, inhibition of ROS by NAC also suppressed MCM-triggered LC3 II accumulation and degradation of NF-κB p65, a cargo of autophagosomes in MCM-polarized macrophages^5^, or p62 (Fig. 1c). These results revealed that generation of ROS is involved in hepatoma-triggered autophagy and M2 macrophage polarization. NOX2 is a membrane-associated enzyme and key producer of ROS in macrophages. Next, we examined whether NOX2 is involved in ROS generation in MCM-treated BMDMs. Gp91^phox^ is a critical component of the NOX2 complex supporting the production of ROS. Therefore, wild type (WT) and gp91^phox−/−^ BMDMs were used for treatment with MCM to determine ROS generation. Compared to WT cells, MCM-induced ROS generation was dramatically decreased in gp91^phox−/−^ BMDMs (Fig. 1d). In addition, reduced production of IL-10 was found in MCM-treated gp91^phox−/−^ BMDMs compared with WT cells (Fig. 1e). Interestingly, the induction of LC3 puncta and LC3-II accumulation by MCM was also abolished in gp91^phox−/−^ BMDMs compared with WT cells (Fig. 1f,g). Furthermore, compared to WT cells, down-regulation of NF-κB p65 and p62 were both recovered in MCM-treated gp91^phox−/−^ BMDMs (Fig. 1g). These data indicate that NOX2-dependent ROS generation regulates hepatoma-induced autophagy and M2 macrophage polarization.

### TLR2 signaling is able to regulate NOX2-dependent ROS generation and M2 macrophage polarization

**Figure 2:**
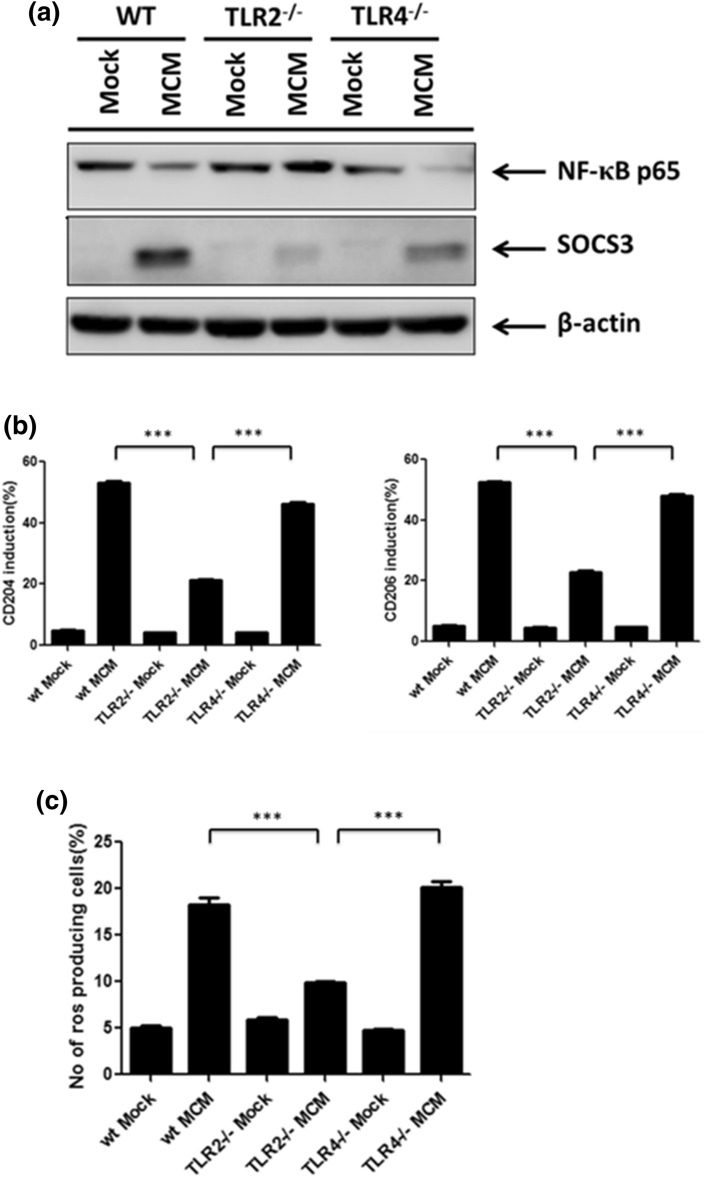
TLR2 is crucial for MCM-induced ROS and M2 macrophage polarization. (**a**) BMDMs from WT, TLR2^−/−^ and TLR4^−/−^ mice were collected and treated with MCM for 24 h. All cell lysates were collected to determine NF-κB p65 and SOCS3 by Western blotting. (**b**) BMDMs from WT, TLR2^−/−^ and TLR4^−/−^ mice were treated with MCM for 24 h to determine the expression CD204 and CD206 by flow cytometry. (**c**) WT, TLR2^−/−^ or TLR4^−/−^ BMDMs were treated with MCM for 12 h. The ROS production of these cells was analyzed by flow cytometry. ***p < 0.0001.

**Figure 3:**
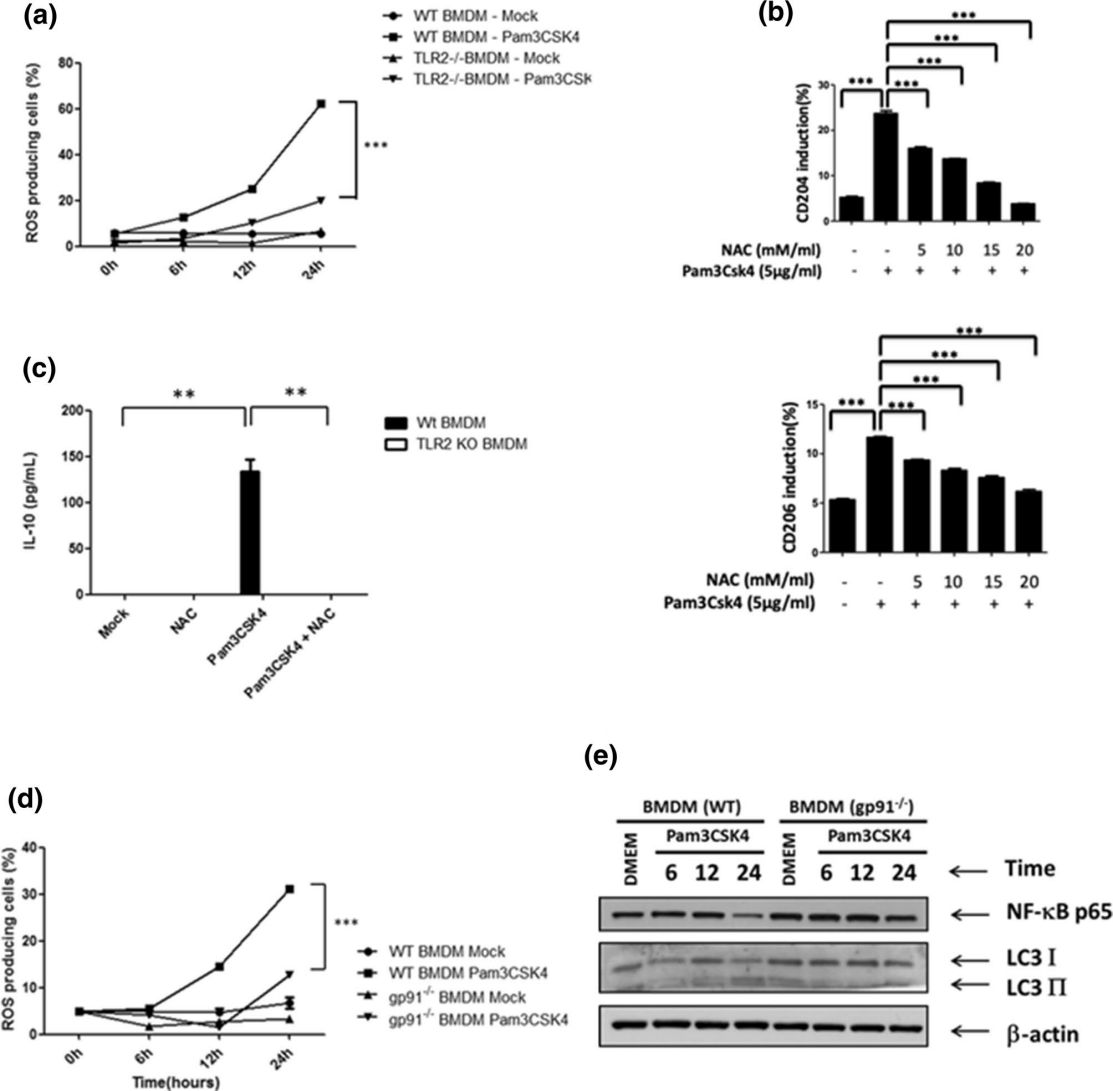
Activation of TLR2 signaling by Pam3CSK4 triggers NOX2-dependent ROS generation. (**a**) WT or TLR2^−/−^ BMDMs were treated with or without Pam3CSK4 (5 μg/ml) for the indicated time and the ROS generation was measured by flow cytometry. (**b**) BMDMs were treated with Pam3CSK4 (5 μg/ml) in the presence or absence of NAC (5,10,15, and 20 mM) for 24 h and the expression of CD204 and CD206 on these cells was analyzed by flow cytometry. (**c**) WT or TLR2^−/−^ BMDMs were treated with Pam3CSK4 (5 μg/ml) in the presence or absence of NAC (10 mM) for 24 h and the production of IL-10 was measured by ELISA. (**d**) WT or gp91^−/−^ BMDMs were treated with or without Pam3CSK4 (5 μg/ml) for the indicated time and the ROS generation was measured by flow cytometry. (**e**) WT or gp91^−/−^ BMDMs were treated with or without Pam3CSK4 (5 μg/ml) for the indicated time. Their cell lysates were then collected to determine the expression of NF-κB p65 and LC3-I/II by Western blotting. *p < 0.05; **p < 0.001; ***p < 0.0001.

Our previous findings showed that the TLR2 signal plays an important role in promoting MCM-driven M2 macrophage polarization. Hence, we next examined whether the TLR2 signal is required for NOX2-dependent ROS generation. Here we showed that the expression of SOCS3, CD204 and CD206 was reduced, whereas the NF-κB p65 level was rescued in MCM-treated TLR2^−/−^ BMDMs compared to WT or TLR4^−/−^ cells (Fig. 2a,b). These results confirmed that TLR2 is crucial in MCM-driven M2 macrophage polarization. Next, we monitored the ROS generation in MCM-treated WT, TLR2^−/−^ and TLR4^−/−^ BMDMs. MCM stimulated high level of ROS production in WT and TLR4^−/−^ BMDMs, which was canceled in TLR2^−/−^ cells (Fig. 2c), suggesting that MCM triggered ROS generation via the TLR2 receptor. To further examine whether the TLR2 signal is able to trigger NOX2-depedent ROS generation, a specific TLR2 agonist, Pam3CSK4, was used. Our results showed that Pam3CSK4 induced increased level of ROS, CD204, CD206 and IL-10 in MCM-treated WT BMDMs, and this was attenuated in TLR2^−/−^ BMDMs or in the presence of NAC (Fig. 3a–c). These results indicate that Pam3CSK4 is able to stimulate ROS production and promotes M2 macrophage polarization. To clarify whether Pam3CSK4 promotes M2 macrophage via NOX2-dependent ROS generation, gp91^phox−/−^ BMDMs were treated with Pam3CSK4. The production of ROS was significantly reduced in Pam3CSK4-stimulated gp91^phox−/−^ BMDMs compared with WT cells (Fig. 3d). Furthermore, the expression of NF-κB p65 was restored, whereas no increased LC3 II accumulation was detected in the Pam3CSK4-stimulated gp91^phox−/−^ BMDMs compared with WT cells (Fig. 3e). These results suggest that TLR2 signaling facilitates M2 macrophage polarization via NOX2-dependent autophagy.

### Hepatoma-derived HMGB1 drives M2 macrophage polarization via TLR2 receptor

**Figure 4:**
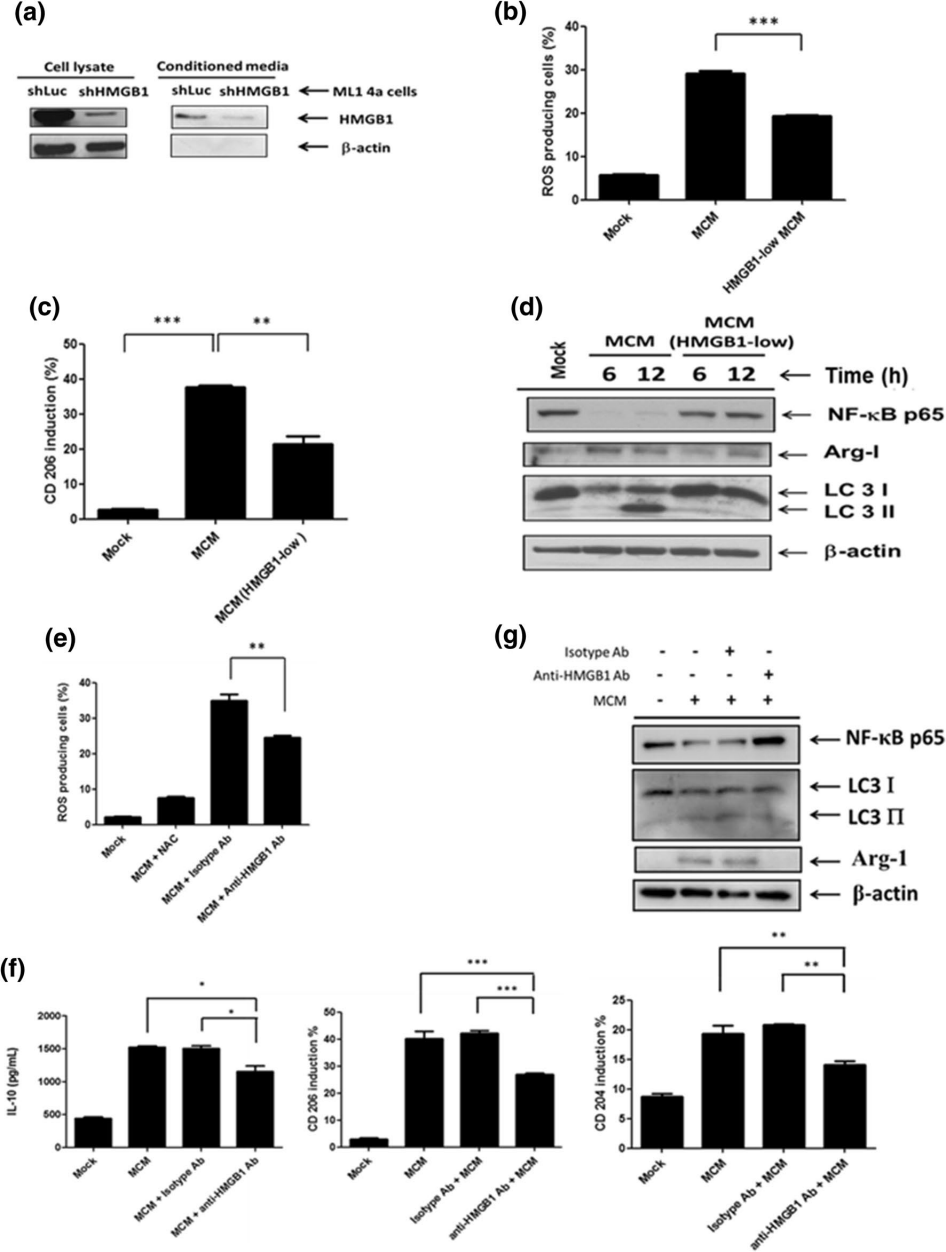
Hepatoma-derived HMGB1 contributes to induce M2 macrophage polarization. (**a**) Cell lysates and conditioned medium from shLuc or shHMGB1 ML-1_4a_ cells were collected and the expression of HMGB1 was determined by Western blotting. (**b**) BMDMs were treated with control or HMGB1-low MCM for 24 h and the ROS production was determined by flow cytometry. (**c**) BMDMs were treated with control or HMGB1-low MCM for 24 h and the CD206 expression was determined by flow cytometry. (**d**) BMDMs were treated with control or HMGB1-low MCM for the indicated time and their cell lysates were collected to determine the expression of NF-κB p65, Arg-1, and LC3-I/II by Western blotting. (**e**) BMDMs were treated with MCM in the presence of isotype or anti-HMGB1 antibodies for 24 h. ROS production was analyzed by flow cytometry. (**f**) BMDMs were treated with MCM in the presence of isotype or anti-HMGB1 antibodies for 24 h to determine the production of IL-10 by ELISA and CD204 or CD206 by flow cytometry. (**g**) BMDMs were treated with MCM in the presence of isotype or anti-HMGB1 antibodies for 24 h and their cell lysates were collected to determine the expression of NF-κB p65, Arg-1 and LC3-I/II by Western blotting. *p < 0.05; ** < 0.001; ***p < 0.0001.

**Figure 5:**
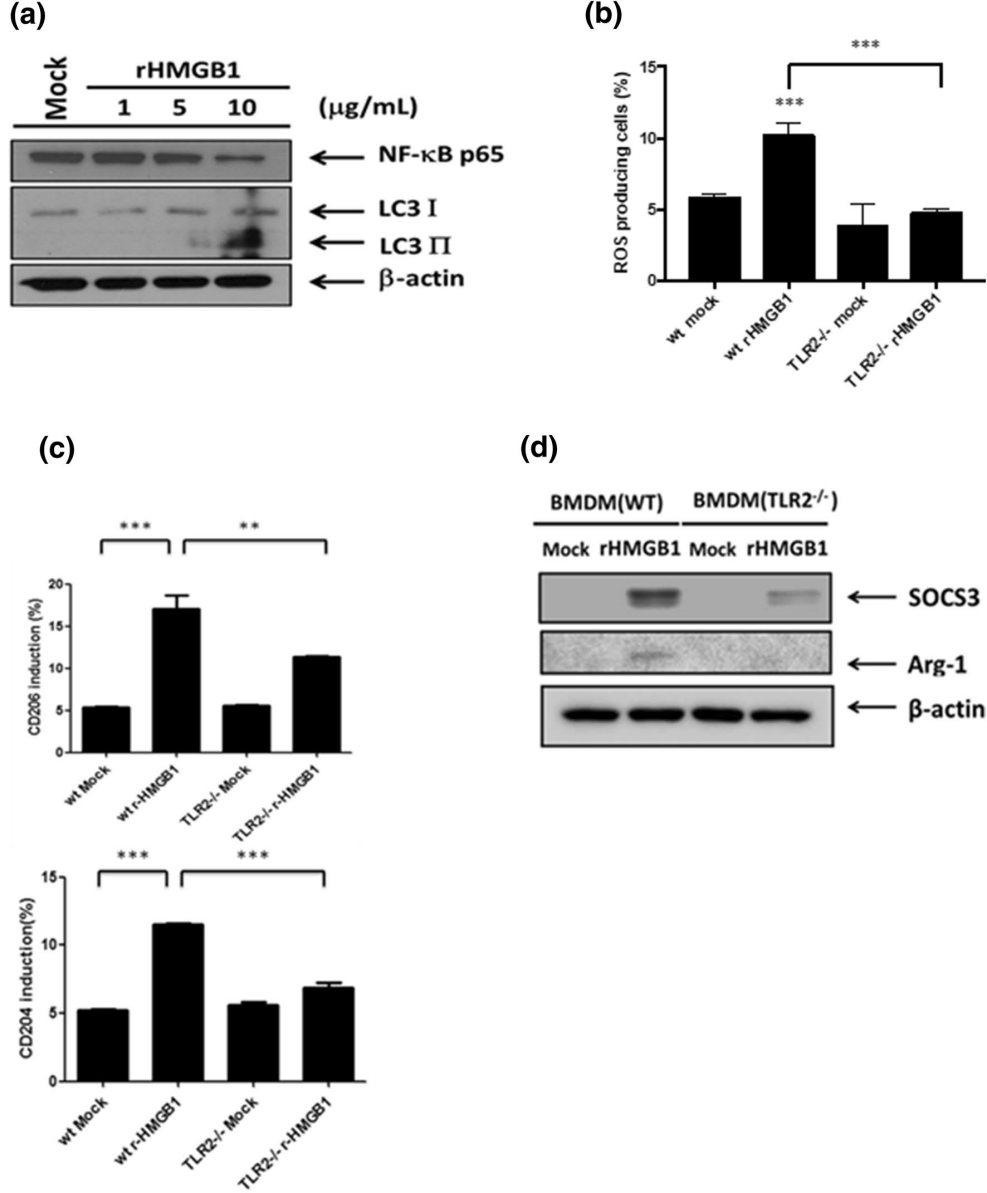
Recombinant HMGB1 triggers autophagy and M2 macrophage polarization via TLR2. (**a**) BMDMs were treated with various concentrations of recombinant HMGB1 (rHMGB-1) for 24 h and their cell lysates were collected to analyze the expression of NF-κB p65 and LC3-I/II by Western blotting. (**b**) WT or TLR2^−/−^ BMDMs were treated with r-HMGB1 (10 μg/ml) for 24 h and the ROS production was analyzed by flow cytometry. (**c**) WT or TLR2^−/−^ BMDMs were treated with r-HMGB1 (10 μg/ml) for 24 h and their surface CD204 and CD206 were analyzed by flow cytometry. (**d**) WT or TLR2^−/−^ BMDMs were treated with rHMGB1 (10 μg/ml) for 24 h and their cell lysates were collected to determine the expression of SOCS3 and Arg-1 by Western blotting. **p < 0.001; ***p < 0.0001.

Next, we went further to investigate how HCC cells polarized M2 macrophage via TLR2 signaling. HMGB1 is able to activate TLR2 signaling and has a potent ability to promote M2 macrophage polarization^14,15^. In addition, HMGB1 has been reported as a pathogenic factor in HCC^16^, and is upregulated in ML-1_4a_ cells compared with non-malignant mouse embryonic fibroblasts (MEF) cells (Supplementary Fig. S1a) and found in MCM (Supplementary Fig. S1a and Fig. 4a). Furthermore, the induced levels of CD204 and CD206 on BMDMs by MEFCM were reduced compared with MCM (Supplementary Fig. S1b). Therefore, we examined whether hepatoma-derived HMGB1 facilitates MCM-triggered M2 macrophage polarization. To test this, we first established HMGB1-silencing ML-1_4a_ cells and collected the low-HMGB1 conditioned medium (Fig. 4a). Then BMDMs were treated with MCM from shLuc- or shHMGB1-ML-1_4a_ cells to monitor the M2 macrophage polarization. The results, shown in Fig. 4b,c, indicate that the MCM-induced up-regulated levels of ROS, CD206, arginase-1, and LC3-II were all inhibited in HMGB1^low^ MCM-treated BMDMs compared with HMGB1^high^ MCM-treated cells. Furthermore, the reduction of NF-κB p65 expression was recovered as well (Fig. 4d). Consistent with these findings, MCM-stimulated up-regulation of ROS, IL-10, CD204, CD206, arginase-1, and LC3-II, as well as reduction of NF-κB p65, was all restored in the presence of neutralizing anti-HMGB1 antibody compared with isotype antibody-treated BMDMs (Fig. 4e–g). These results indicate that blockage of hepatoma-cell-derived soluble HMGB1 inhibits MCM-triggered M2 macrophage polarization. To further confirm the regulatory activity of HMGB1 on M2 macrophage polarization, BMDMs were stimulated with mouse recombinant HMGB1 (rHMGB1). We found that NF-κB p65 degradation and LC3-II accumulation were induced in rHMGB1-treated BMDMs (Fig. 5a). In addition, treatment of rHMGB1 triggered production of ROS, CD204, CD206, arginase-1 and SOCS3 in WT BMDMs, which were all attenuated in TLR2^−/−^ BMDMs (Fig. 5b–d). These results indicate that hepatoma-derived HMGB1 may facilitate ROS-mediated M2 macrophage polarization via the TLR2 receptor.

### Blockage of HMGB1 and ROS reduces amounts of tumor-associated M2 macrophages and attenuates hepatoma growth in mice

**Figure 6:**
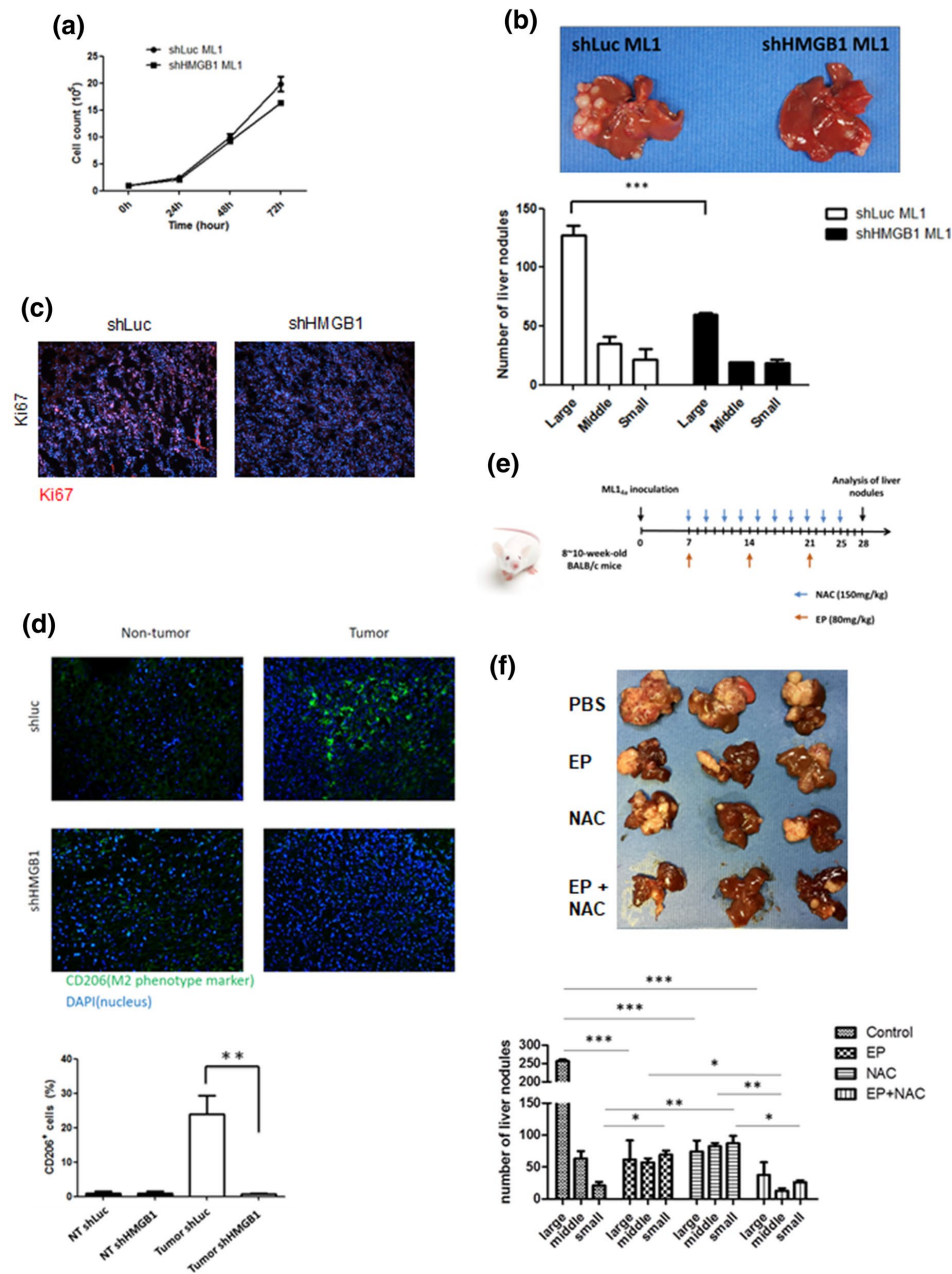
Blockage of HMGB1 and ROS reduces amounts of tumor-associated M2 macrophages and attenuates hepatoma growth in mice. (**a**) Cell growth of shLuc and shHMGB1 ML-1_4a_ cells was monitored for 72 h. (**b**,**c**) BALB/c mice (n = 5) were intrasplenically injected with shLuc or shHMGB1 ML-1_4a_ cells. After 28 days post injection, all mice were sacrificed, and their livers were removed to quantify the numbers and sizes of nodules (**b**). Parts of these livers were also sectioned to stain with Ki67 or anti-CD206 antibodies (**c**,**d**). (**e**,**f**) Schematic diagram of treatment protocol. 8 to 10 week old BALB/c mice (n = 5) were intrasplenically inoculated with ML-1_4a_ cells to establish the liver nodule formation. NAC (150 mg/kg) was given to the mice intraperitoneally at two-day intervals beginning on day 7 post tumor injection. EP (80 mg/kg) was given to mice intraperitoneally at seven-day intervals beginning on day 7 post tumor injection. On day 28 post tumor injection, all treated-mice were sacrificed and their livers were removed to quantify the numbers and sizes of nodules (**e**). Results were quantified from 3 independent experiments. * p < 0.05; ** < 0.001; ***p < 0.0001.

We then went further to examine the regulatory role of hepatoma-derived HMGB1 in hepatoma-bearing mouse. To understand whether HMGB1 may have intrinsic effects on hepatoma cell growth, we first monitored the cell growth of shLuc- and shHMGB1-ML-1_4a_ cells in vitro. The results showed no significant difference in cell growth between shLuc- and shHMGB1-ML-1_4a_ cells in vitro (Fig. 6a). Then we used a murine in situ hepatoma model, which was set up by intrasplenic injection of 10^6^ shLuc- or shHMGB1-ML-1_4a_ cells to mice, to examine the extrinsic role of HMGB1 on hepatoma growth. Interestingly, the sizes and numbers of tumor nodules were significantly decreased in shHMGB1-ML-1_4a_-bearing mice compared with shLuc control groups (Fig. 6b). By Ki67 immuno-fluorescence staining assay, the expression levels of Ki67 in shLuc tumors were much higher than shHMGB1 tumors, indicating that loss of HMGB1 reduces hepatoma proliferation in vivo (Fig. 6c). In addition, we detected CD206-expressed cells in shLuc- and shHMGB1- ML-1_4a_ tissues. Compared to non-tumor tissues, increased CD206 + cells were clearly observed in shLuc- ML-1_4a_ tissues, indicating that M2-macrophages were accumulated within hepatoma. However, significant reduction of CD206 + cells in shHMGB1-hepatoma tissues was found compared with shLuc groups (Fig. 6d). This indicates that hepatoma-derived HMGB1 promotes tumor-associated M2 macrophage accumulation and tumor growth in hepatoma-bearing mice. Next, we tested whether blockage of HMGB1, ROS or both of them is able to reduce liver nodule formation in mice. To examine this, the inhibitor of HMGB1, ethyl pyruvate (EP), and the inhibitor of ROS, NAC, were administrated individually or together to hepatoma-bearing mice (Fig. 6e). According to the results in Fig. 6e, treatment of EP or NAC alone decreased liver nodule formation in hepatoma-bearing mice compared with PBS-treated control groups. Notably, the combined treatment of EP and NAC showed the most significant reduction in numbers and sizes of liver nodules in hepatoma-bearing mice compared with the PBS, EP or NAC single treatment groups (Fig. 6f). Taking these results together, we have demonstrated that hepatoma-derived HMGB1 facilitates M2 macrophage recruitment and tumor growth while blockage of HMGB1 and ROS inhibits hepatoma growth in mice.

## Discussion

**Figure 7:**
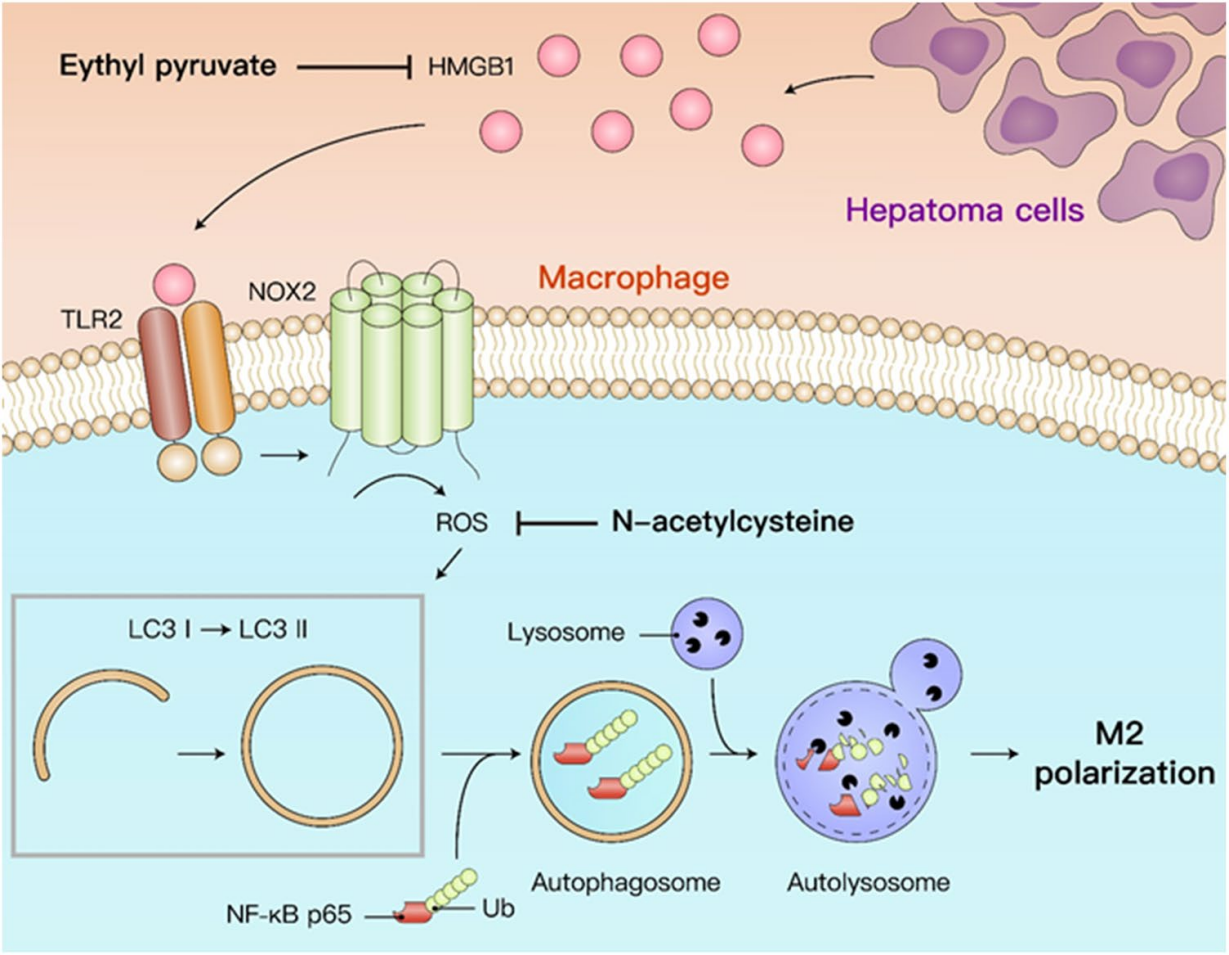
Schematic diagram of HMGB1-induced M2 macrophage polarization.

In this study, we investigated the mechanisms of autophagy-regulated M2 macrophage polarization induced by hepatoma cells. Hepatoma-derived HMGB1 stimulates NOX2-dependent ROS generation via TLR2 to trigger autophagy formation, which leads to lysosomal degradation of NF-κB p65 and hence maintains the M2 macrophage polarization. Blockage of ROS and HMGB1 is able to reduce accumulation of tumor-associated M2 macrophages and attenuate the hepatoma growth in mice. Here, we uncovered a new regulatory mechanism of the HMGB1-triggered TLR2/NOX2/autophagy axis in hepatoma-prompted M2 macrophage polarization (Fig. 7).

NOX-derived ROS production is reported to control M1/M2 macrophage polarization in regulating inflammation, infection and tumor growth. Increased ROS in M1 macrophages is believed to enhance phagocytic activity and trigger pro-inflammatory responses, which is beneficial in the defense against bacterial invasion^17^. Interestingly, emerging studies have revealed that this NOX-generated ROS is also responsible for M2 macrophage polarization, particularly in tumor microenvironments^8,18^. NOX1/NOX2 deficiency mice showed decreased ROS production in TAM and impaired M2 differentiation, leading to tumor growth inhibition. Inactivation of JNK and ERK in NOX1/NOX2 deficient macrophages is suggested to mediate this alteration^9^. How NOX-generated ROS and JNK/ERK facilitate M2 macrophage polarization is so far not clear. Here, we found that NOX2-dependent ROS is able to trigger autophagy to down-regulate NF-κB p65, which promotes hepatoma-induced M2 macrophage differentiation. In addition, we have previously demonstrated that ERK activation is required for this lysosomal degradation of NF-κB p65 in hepatoma-polarized M2 macrophages^5^. According to these observations, we suggest that NOX-generated ROS regulates M2 macrophage polarization partly by up-regulating autophagic and lysosomal activities. Furthermore, to understand the involvement of NOX1 in MCM-induced M2 macrophage polarization, the NOX1 specific inhibitor ML171 was used. According to our results, the MCM-induced ROS production, CD204 and CD206 on BMDMs were all attenuated by ML171. This suggests that both NOX1 and NOX2 are crucial for MCM-induced M2 macrophage polarization (Supplementary Fig. S2). In the future, the genetic NOX1 deficient macrophages should be used to confirm the role of NOX1 in this phenomenon. On the other hand, a recent study revealed that induction of mitochondrial ROS (mtROS) triggers a functional M2 macrophage polarization to protect intestinal inflammation^19^. mtROS is known as an inducer of autophagy^20^, so the role of mtROS in hepatoma promoted M2 macrophage polarization needs to be further examined. Earlier studies have demonstrated that the recruited TAM in tumor microenvironments may come from tissue resident macrophages and BMDMs^21^. To examine whether liver Kupffer cells are recruited and express M2-phenotye in ML-1_4a_ tumors, we co-stained CLEC4F, which is considered as a marker of mouse Kupffer cells^22^, with CD206 in tumor area. According the results were showed in Supplementary Fig. S3, CLEC4F + Kupffer cells were found in both tumor and non-tumor areas. However, these CLEC4F + Kupffer cells did not express CD206. This result suggests that liver Kupffer cells may not response to ML-1_4a_-triggered M2 macrophage polarization.

The functions of HMGB1 in HCC are complicated due to its different intracellular and extracellular locations. Intracellular HMGB1 has been shown to control cell cycle, survival, proliferation, and differentiation of HCC^23^. The post-translational modification of HMGB1, including acetylation and redox modification, is able to trigger different bioactivities of HMGB1^24^. Acetylation of HMGB1 is reported to help HMGB1 to leave the nucleus to enter the cytoplasm where it is able to be packaged into specialized secretory vesicles, then be released into the extracellular environment^25^. The redox modification of secreted HMGB1 in three cysteine residues (at positions 23, 45, and 106) is found to regulate its receptor binding^24^. HMGB1 is a kind of DAMPs when it is secreted out of cells. As a danger signal molecule, secreted HMGB1 will trigger outside-in signaling cascades to regulate inflammation, cell differentiation, cell migration, and tumor metastasis via binding to several identified membrane receptors^26^. Interestingly, this location-driven function of HMGB1 is also reported in HMGB1-induced autophagy. In the nucleus, HMGB1 is able to act as a transcriptional factor to increase the transcription of heat-shock protein 27 and lead to induction of autophagy via the PINK1/Parkin-dependent pathway^27,28^. In the cytoplasm, HMGB1 promotes phosphorylation of Bcl-2 to dissociate Beclin-1/Bcl2 complex, which in turn facilitates formation of Beclin-1/PI3K complex to initiate autophagy^29^. In the extracellular environment, secreted HMGB1 triggers ERK and AMPK/mTOR pathways to activate autophagy by binding its membrane receptors^30^. The secreted HMGB1-induced autophagy has been reported to be beneficial for cancer progression. By binding with RAGE on HCC cells, secreted HMGB1-triggered autophagy is able to facilitate HCC cell proliferation and resistance to sorafenib-induced cytotoxicity^31^. In Fig. 6a, there is no difference in cell proliferation between shLuc and shHMGB1 ML-1_4a_ cells. Since the expression levels of RAGE on HCC cells corelate to the HMGB1-regulated cell proliferation^31^, the expression levels of RAGE on ML-1_4a_ cells might not be upregulated. Here we further demonstrated that HMGB1-induced autophagy contributes to TAM M2 macrophage polarization in the HCC microenvironment. These findings indicate HMGB1 as a novel target for the treatment of HCC. On the other hand, as results were showed in Fig. 4, both HMGB1-low MCM and neutralizing antibodies against HMGB1 reduced partly MCM-induced effects on BMDM. This indicates that HMGB1 is not the only one factor in MCM to affect the M2 macrophage polarization. Further investigation to identify other potential factors derived from HCC cells to drive M2 macrophage polarization is needed.

Activation of TLR signaling on macrophages has been suggested to be involved in the induction of tumor-associated inflammatory responses. DAMPs from dying tumor cells, such as heat shock proteins or HMGB1, are able to trigger protective immune responses against the tumor growth via TLR signaling^32,33^. However, recent reports have suggested that TLR-mediated activation on macrophages may promote tumor development. For example, activation of TLR2 or TLR4 signals on TAMs increases the lung cancer metastasis via TNF-α and IL-6 production^34,35^. A TLR9/IL-6/JAK/STAT3 pathway in tumor-associated myeloid cells plays an essential role in initiating the cancer recurrence after radiotherapy^36^. The tumor-derived intermediate-sized hyaluronan fragments have been recently reported to induce M2 macrophage polarization via the TLR4/mir-935 pathway to promote tumor progression^37^. A lung cancer-derived extracellular-matrix protein versican is reported to cause a TLR2-dependent activation and M2-phenotype polarization in TAM to induce tumor metastasis^38^. In addition, tumor-derived exosome-containing heat-shock protein 72 is able to trigger the immunosuppressive function of tumor-associated myeloid cells via the TLR2/MyD88 pathway^39^. Similar to these observations, we previously reported that TLR2-dependent autophagy is necessary to regulate the HCC-associated M2 macrophage polarization^5^ and further identified the HCC-derived soluble factor HMGB1 in this study. Our findings are consistent with a recent study demonstrating that tumor-derived exosomal HMGB1 promotes the progression of esophageal squamous cell carcinoma through inducing expansion of PD1^+^ M2-phenotype TAM^12^. However, we did not observe the complete reduced levels of CD204, CD206 and ROS in MCM-treated TLR2^−/−^ BMDM compared with wild type cells. This indicates that there are other signals from MCM to control this M2 macrophage polarization. In this study, we further showed that NOX2-produced ROS is involved in this TLR2-dependent M2 macrophage polarization. Some research findings point to NOX2 as an important mediator for TLR2-regulated immune responses. In particular, the interaction between NOX2 and TLR2 has been suggested to facilitate in the defense against mycobacteria infection by inducing cathelicidin expression^40^. Also, the requirement of NOX2 in TLR2/6-mediated GM-CSF production from endothelial cells or TLR2-depenedent nerve injury via microglia cells has been noted^41,42^. However, the mechanism of TLR2-generated ROS via NOX2 has not yet been clearly defined. A previous report indicated that formation of TLR2/MyD88/TRAF6/c-Src/NADPH oxidase complex is crucial for lipoteichoic acid-induced ROS production in human tracheal smooth muscle cells^43^. Further study would be worthwhile to examine whether this complex is formed and is responsible for the HMGB1-triggered M2 macrophage polarization.

## Materials and methods

### Reagents and antibodies

Pam3CSK4 was purchased from InvivoGen (CA, USA). Recombinant murine HMGB1 was purchased from eBioscience (CA, USA). 2-acetylphenothiazine (ML171) was purchased from Sigma-Aldrich (MO, USA). Antibodies against LC3 and p62 were purchased from MBL (Nagoya, Japan). Antibodies against NF-κB p65, arginase 1 and ERK (p44/42) were purchased from Cell Signaling Technology (MA, USA). Anti-mouse CD204-FITC and CD206-FITC antibodies were purchased from AbD Serotec (Oxford, UK) and Biolegend (CA, USA), respectively. Antibodies against β-actin and HMGB1 were purchased from Abcam (MA, USA). Antibodies against SOCS3 were purchased from Proteintech (IL, USA). Anti-HMGB1 neutralized chicken IgY antibodies were purchased from SHINO-TEST Corporation (Kanagawa, Japan), and isotype chicken IgY antibodies were purchased from BD Biosciences (CA, USA). Antibodies against Ki67 and CLEC4F/CLECSF13 as well as mice cytokine ELISA kits were purchased from R&D systems (MN, USA).

### Cell culture

Human embryonic kidney cells 293 T were purchased from American Type Culture Collection Cell bank. Mouse hepatoma cell line ML-1_4a_ cells in BALB/c background were generated previously^44^. Both 293 T and ML-1_4a_ cells were cultured in Dulbecco’s modified Eagle’s medium (DMEM) supplemented with 10% heat-inactivated fetal bovine serum (FBS), 50 U/mL penicillin, and 0.05 mg/ml streptomycin. To culture bone marrow-derived macrophages (BMDMs), two to three 6 to 8-week-old mice were scarified to obtain femurs and tibias of both legs. These bones were sterilized with 75% ethanol and then the ends of the bones were removed. The bone marrow cells from all these bones were flushed out and maintained in RPMI 1640 medium supplemented with 10% FBS. Pooled bone marrow cells (2 × 10^6^) were further cultured in differentiation medium (10% FBS, 0.01 μg/mL MCSF in RPMI 1640) for 6 days.

### Mice in situ hepatoma model

BALB/c (male, 8–10 weeks old) and C57BL/6 mice (female, 8–10 weeks old) were purchased from the Animal Laboratory of National Cheng Kung University. TLR2^−/−^ mice were kindly provided by Dr. John T. Kung (Institute of Molecular Biology, Academia Sinica, Taiwan). TLR4^−/−^ mice were kindly provided by Dr. Akira Shizuo (Laboratory of Host Defense, WPI Immunology Frontier Research Center, Osaka University). gp91 ^phox −/−^ mice were kindly provided by Dr. Chi-Chang Shieh (Institute of Clinical Medicine, National Cheng Kung University, Taiwan). All mice were maintained in the pathogen-free facility of the Animal Laboratory of National Cheng Kung University. The animals were raised and cared for according to the guidelines set up by the Institutional Animal Care and Use Committee (IACUC) of National Cheng Kung University. This study was approved by the Committee on the Ethics of Animal Experiments of National Cheng Kung University (Permit Number: 102117). A murine *in situ* hepatoma model was set up by intrasplenic injection of 1 × 10^6^ viable sh-luciferase (Luc)- or shHMGB1-ML-1_4a_ cells in 0.1 ml of DMEM into anesthetized mice as previously reported^44^. The liver tumor nodules were formed in various sizes beginning 1 week after injection. At 28 to 35 days post tumor cell inoculation, the livers of hepatoma-bearing mice were collected to determine the numbers and sizes of the liver nodules.

### Western blotting

To collect protein extracts, cells were harvested and suspended in lysis buffer (Cell Signaling) on ice for 20 min, and then centrifuged at 12,000 × *g* for another 20 min. The supernatants were collected to determine concentration of proteins. 25 to 30 μg of each protein extract was separated by SDS-PAGE and transferred to PVDF membranes. For detection of proteins, primary antibodies were added to membranes. After incubation with peroxidase-conjugated secondary antibodies, the blots were visualized by enhancing chemiluminescence reagents (PerkinElimer Life Sciences, MA, USA).

### Immunostaining

For cell surface immunostaining, BMDMs were harvested and incubated with fluorescence conjugated antibodies, including FITC-conjugated anti-CD80, CD204, CD206 and PE-conjugated anti- MHC II antibodies, in staining buffer (2% FBS + 0.1% sodium azide dissolved in PBS) for 30 min on ice. The expression of these surface molecules was determined by flow cytometry. To monitor autophagosome formation, cells were fixed with 4% formaldehyde and stained with anti-LC3 antibody at 4 °C. The LC3 puncta formation was determined by a confocal fluorescence microscope (Olympus FV 1,000, Japan).

### ELISA

To determine the M1 or M2 macrophage polarization, BMDMs (3 × 10^4^) were seeded in a 96-well plate and treated with MCM or recombinant HMGB1 for 24 h. Next, the supernatants of these cells were collected. The level of produced cytokines was analyzed by ELISA kits (R&D system Inc, MN, USA).

### Measurement of intracellular ROS

To analyze the ROS production in BMDMs, the cells were incubated with 5 μM 2′, 7′-dichlorodihydrofluorescein diacetate (H*2*DCF-DA) fluorescent probe for 1 h at room temperature. The level of ROS-converted fluorescent 2′,7′-dichlorofluoresce in (DCF) was detected by flow cytometry.

### Lentivirus-based short hairpin RNA (shRNA) transfection

HMGB1 was knocked-down in ML1_4a_ cells by stably expressing lentivirus-based shRNA. The clones were obtained from the National RNAi Core Facility (Institute of Molecular Biology/Genomic Research Center, Academia Sinica, Taiwan) and the target sequence for HMGB1 is TRCN0000365913 5′-GAAGATGATGATGATGAATAA- 3′. To produce recombinant lentivirus**,** 293 T cells (2 × 10^5^) were seeded into a 6 well-plate and transfected with DNA mixture (pCMVdeltaR8.91: 0.9 μg/well; pMD.G: 0.1 μg/well; shRNA: 1 μg/well). ML1_4a_ cells were then infected with recombinant lentivirus for 24 h, and stably expressed cells were selected by puromycin. The knockdown efficiency of target proteins was determined by Western blotting.

### Statistical analysis

All experiments were performed at least two to three times and the data are expressed as means ± SD. Data were analyzed by Two-Way ANOVA using GraphPad Prism 5 software (GraphPad Software Inc., La Jolla, CA, USA) and p < 0.05 was considered to represent significant differences between groups. All experiments were repeated in two to three times.

## Supplementary information

Supplementary information

## Data Availability

The data that support the findings of the study are available from the corresponding author upon request.
