# Supplementary material for: Hepatocellular carcinoma-derived high mobility group box 1 triggers M2 macrophage polarization via a TLR2/NOX2/autophagy axis
Source: Sci Rep. 2020 Aug 12;10:13582. doi: 10.1038/s41598-020-70137-4 (PMC7423894; doi:10.1038/s41598-020-70137-4)
Supplement: Supplementary file 1 — Supplementary information [file 41598_2020_70137_MOESM1_ESM.docx]

**Title:**

Hepatocellular carcinoma-derived high mobility group box 1 triggers M2 macrophage polarization via a TLR2/NOX2/autophagy axis

**Authors**

Dong-Jer Shiau ^1Δ^, Wan-Ting Kuo^2Δ^, Goutham Venkata Naga Davuluri^2^, Chi-Chang Shieh^3,4^, Pei-Jane Tsai^2,5^, Chien-Chin Chen^6,7^, Yee-Shin Lin^1,2,8^, Yi-Zhen Wu^1^, Yu-Peng Hsiao^1^ and Chih-Peng Chang^1,2,8,^*

^1^ Department of Microbiology & Immunology, College of Medicine, National Cheng Kung University, Tainan 701, Taiwan

^2^ The Institute of Basic Medical Sciences, College of Medicine, National Cheng Kung University, Tainan 701, Taiwan

^3^Institute of Clinical Medicine, National Cheng Kung University Hospital, Tainan 701, Taiwan

^4^Division of Allergy, Immunology and Rheumatology, Department of Pediatrics, College of Medicine, National Cheng Kung University Hospital, Tainan 701, Taiwan.

^5^Department of Medical Laboratory Science and Biotechnology, National

Cheng Kung University, Tainan 701, Taiwan

^6^Department of Pathology, Chia-Yi Christian Hospital, Chiayi 600, Taiwan

^7^Department of Cosmetic Science, Chia Nan University of Pharmacy and Science, Tainan 701, Taiwan

^8^ Center of Infectious Disease and Signaling Research, National Cheng Kung University, Tainan 701, Taiwan

ΔThese authors contributed equally to this work

*Corresponding author

Correspondence and requests for materials should be addressed to Chih-Peng Chang

(e-mail: [cpchang@mail.ncku.edu.tw](mailto:cpchang@mail.ncku.edu.tw))

Supplementary Figure 1. HMGB1 is upregulated in ML-1_4a_ cells. (**a**) Cell lysates and supernatants of ML-1_4a_ and MEF cells were collected. The expression of HMGB1 was detected by Western blot. (**b**) BMDMs were treated with MCM or MEFCM for 24 hours and expression of CD206 and CD204 was analyzed by flow cytometry. ***p<0.0001.

Supplementary Figure 2. NOX1 is involved in MCM-regulated M2 macrophage polarization. BMDMs were pretreated with N-acetyl cysteine (ML171, 10 μM) for 30 min prior to adding MCM. Cells were then collected after 24 hours to determine the expression of ROS, CD204 and CD206 by flow cytometry. ***p<0.0001.

Supplementary Figure 3. CLEC4F+ Kupffer cells do not express CD206 in ML-1_4a_ tumors. The ML-1_4a_ cells were intrasplenically injected into BABL/c mice and the livers were then collected at day 28 after tumor inoculation. The livers were sectioned and stained with anti-CLEC4F/CLECSF13 and anti-CD206 antibodies. T: tumor; NT: non-tumor


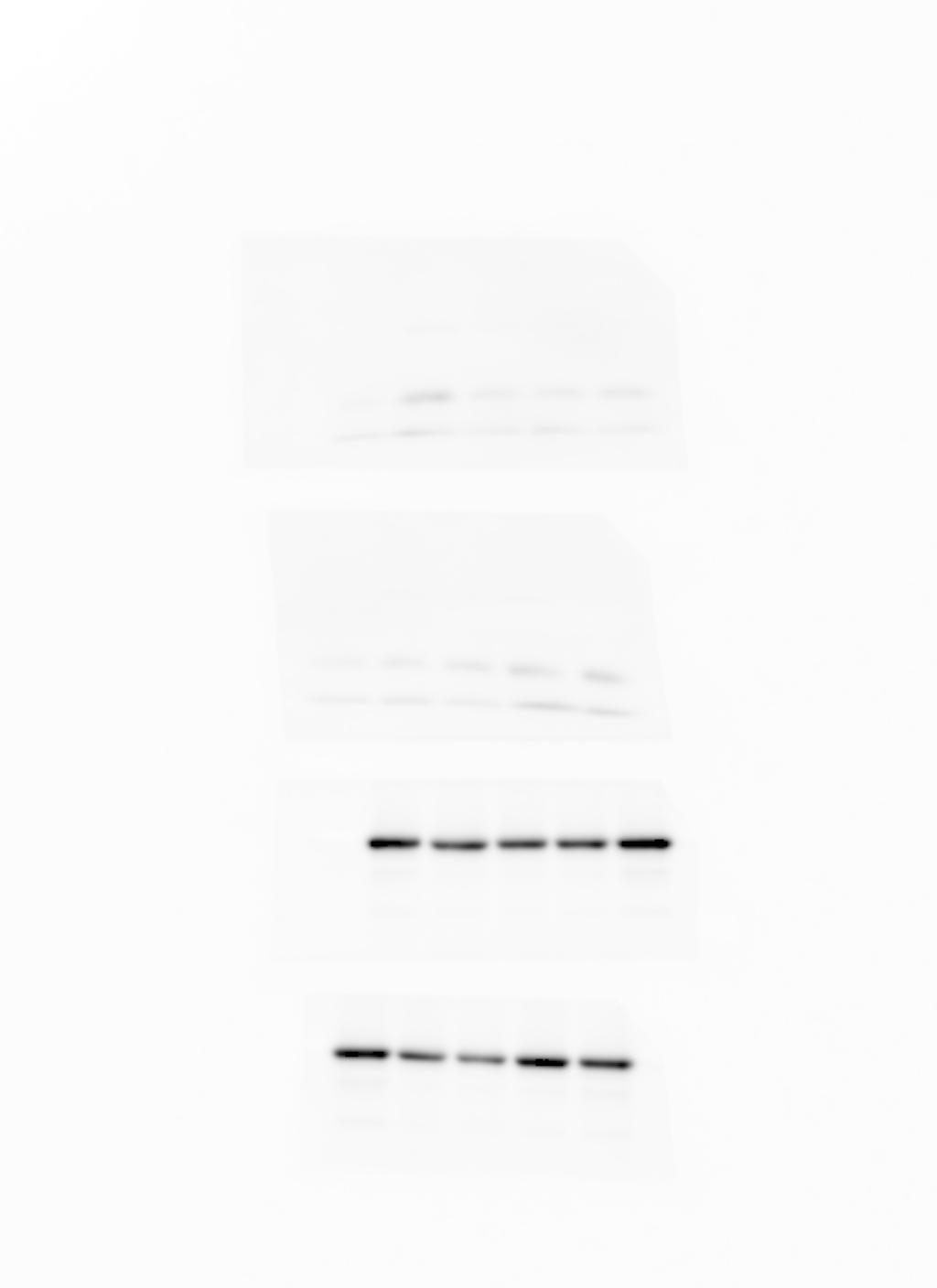

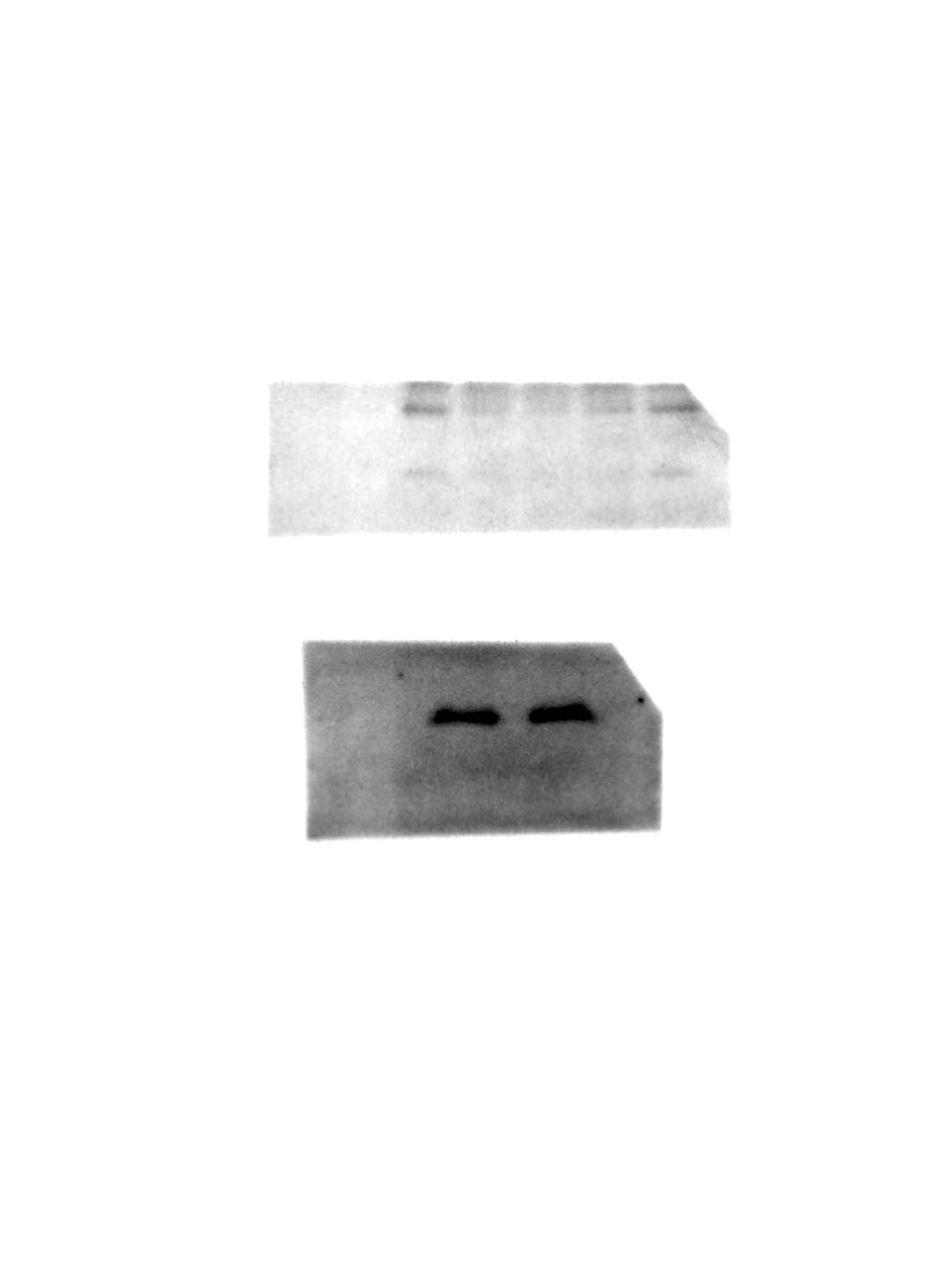

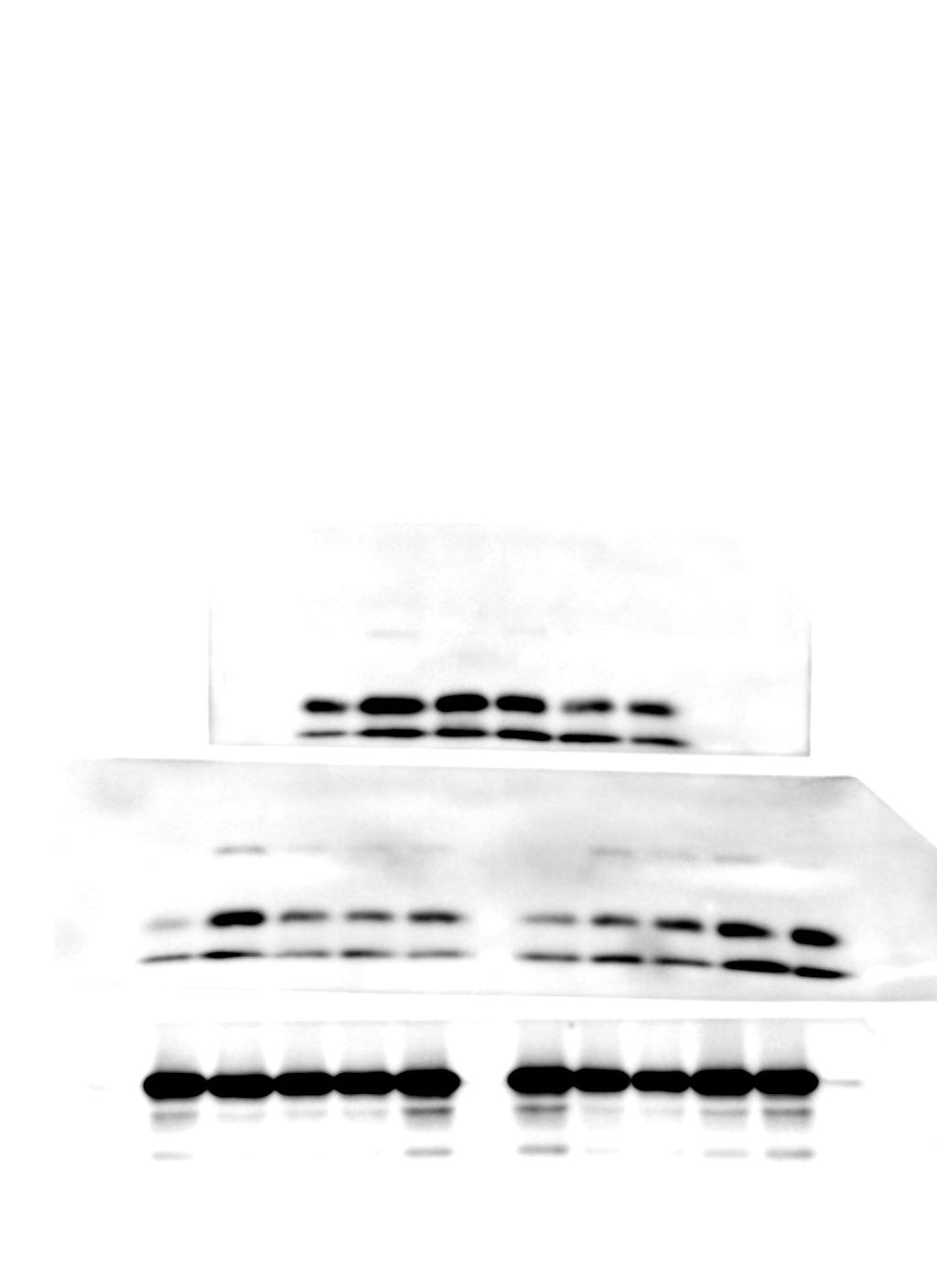

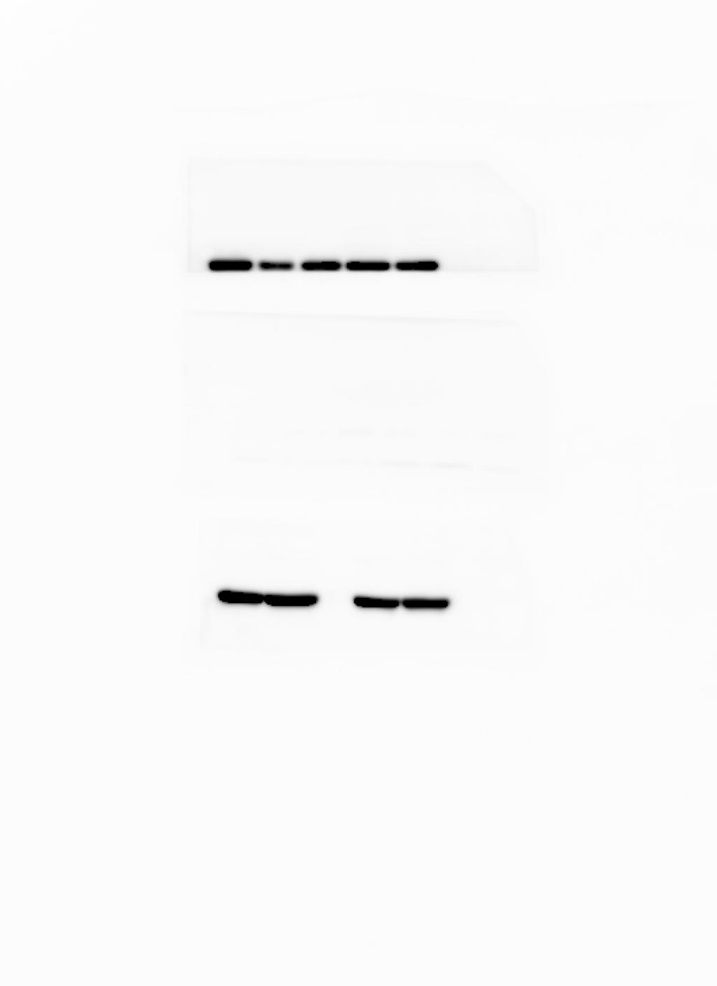


**Original images of immunoblots**

**Fig. 1c**

**Mock**

**MCM**

**1**

**5**

**10**

**(mM)**

**NAC+MCM**

**(Kd)**

**70**

**70**

**15**

**10**

**30**

**NFκB**

**p62**

**LC3I**

**LC3II**

**β-actin**


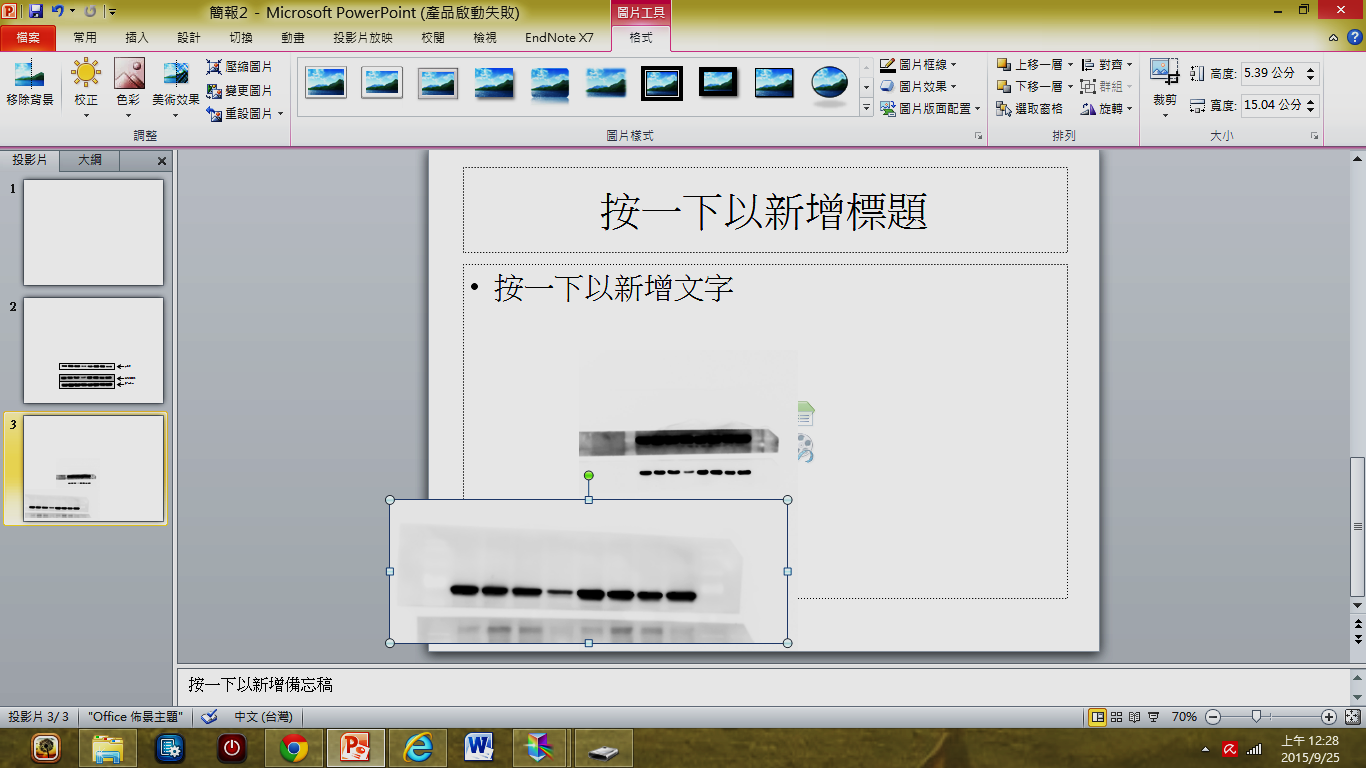


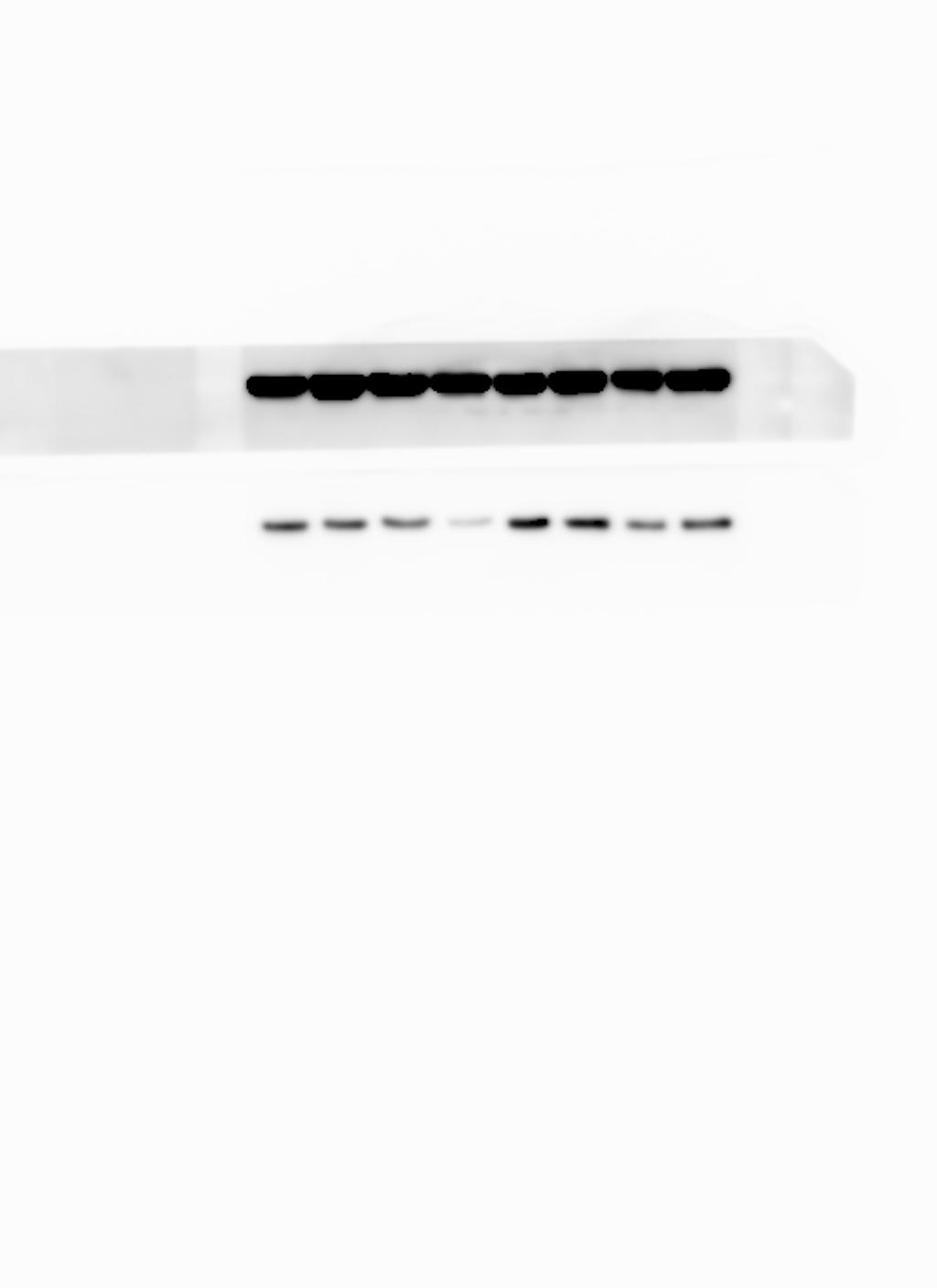

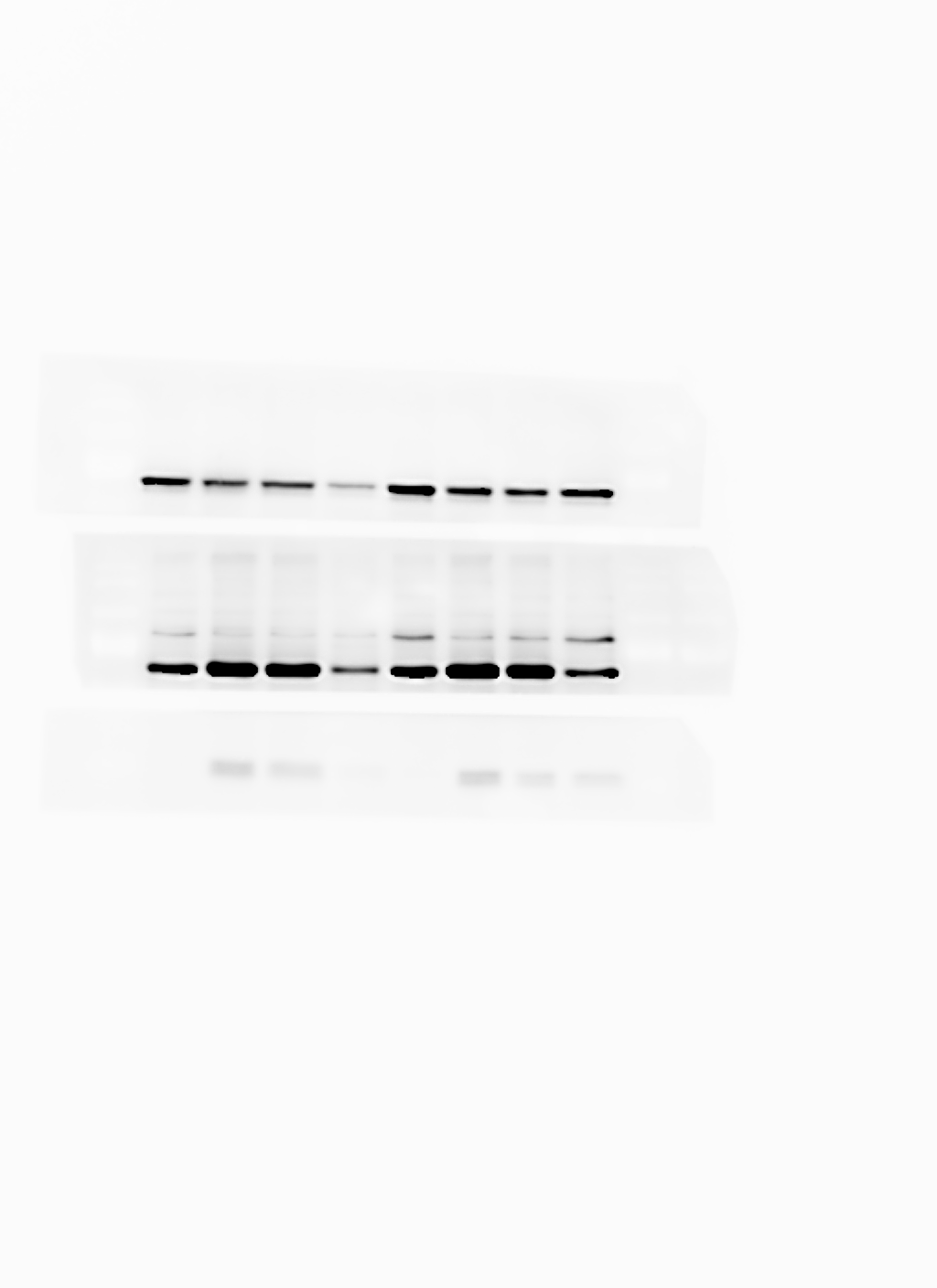

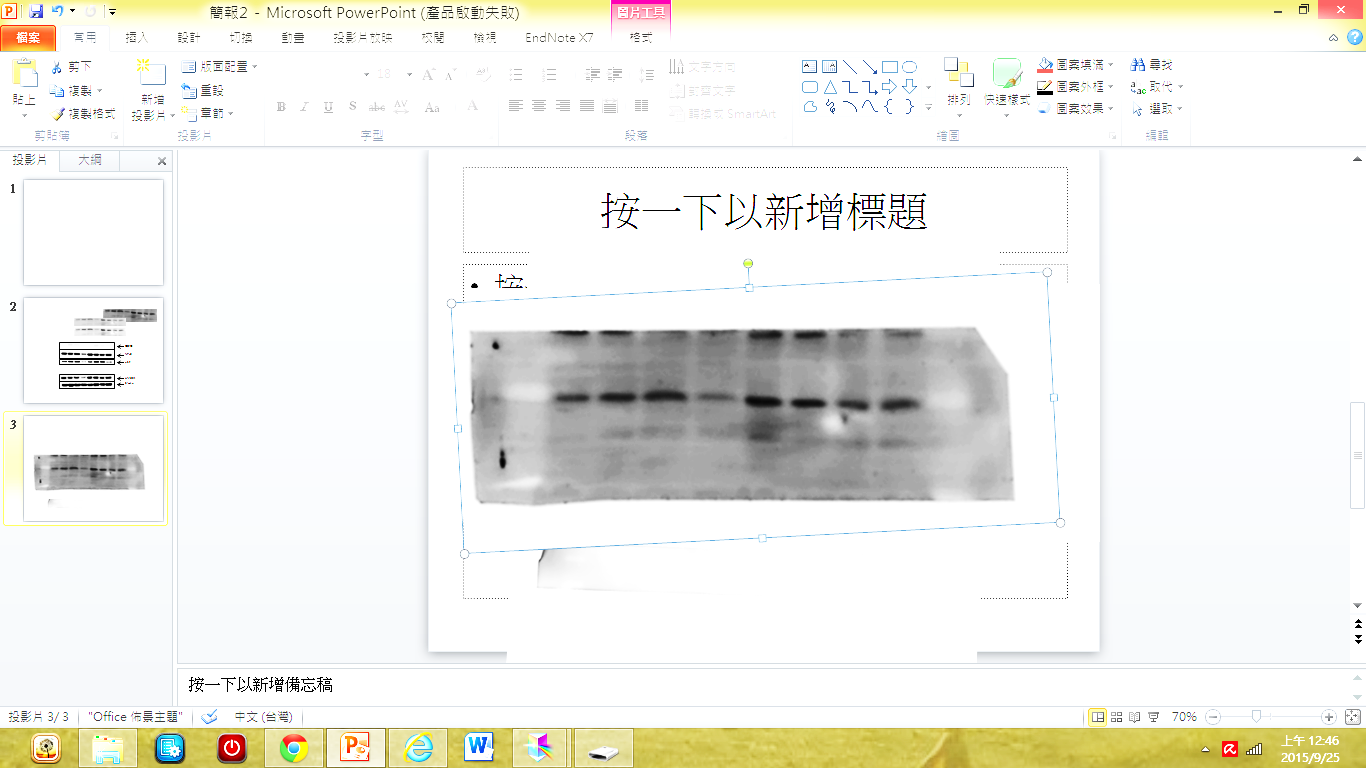


**NFκB**

**p62**

**LC3I**

**LC3II**

**β-actin**

**(Kd)**

**70**

**70**

**15**

**10**

**30**

**Original images of immunoblots**

**Fig. 1g**


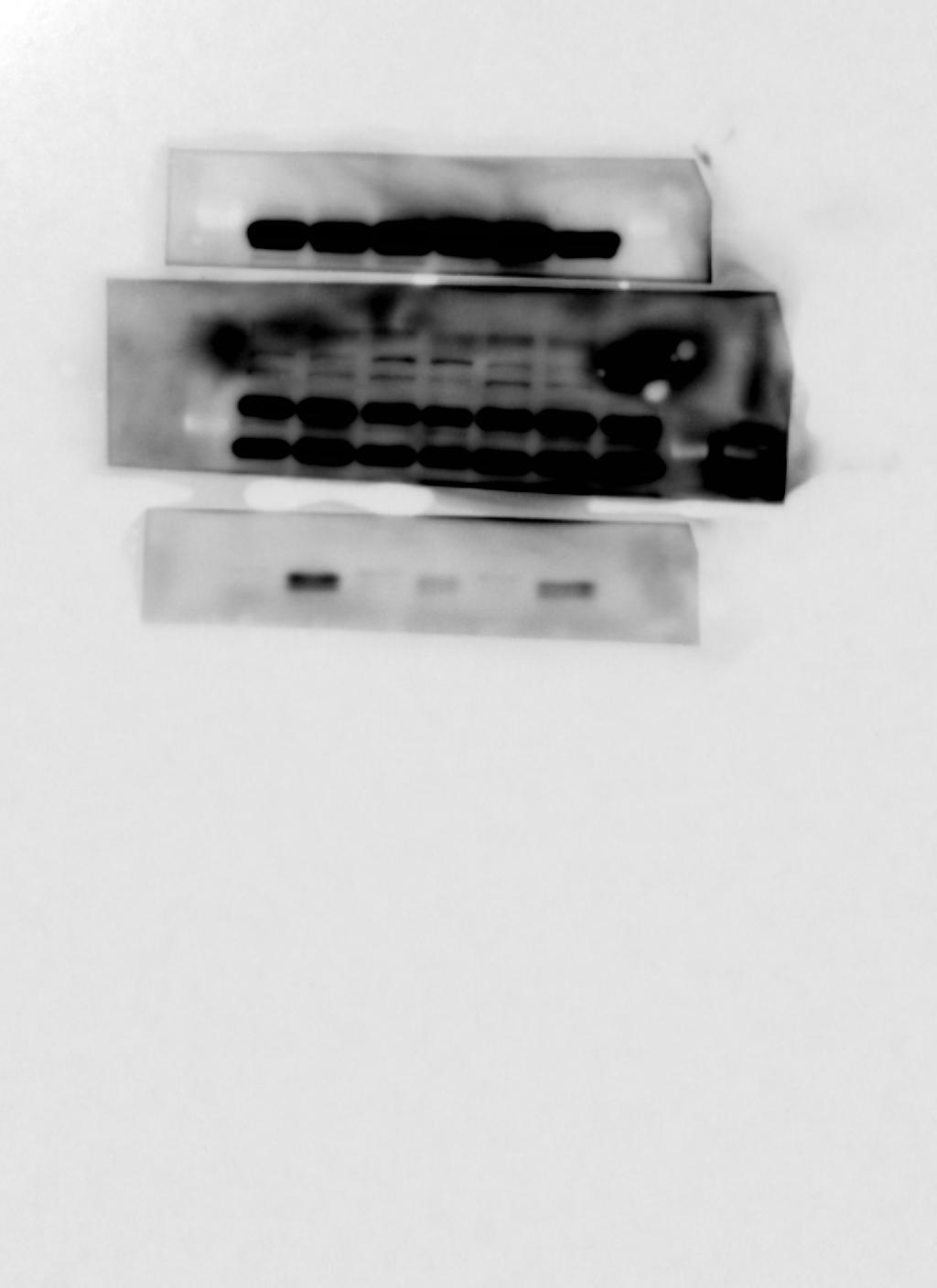

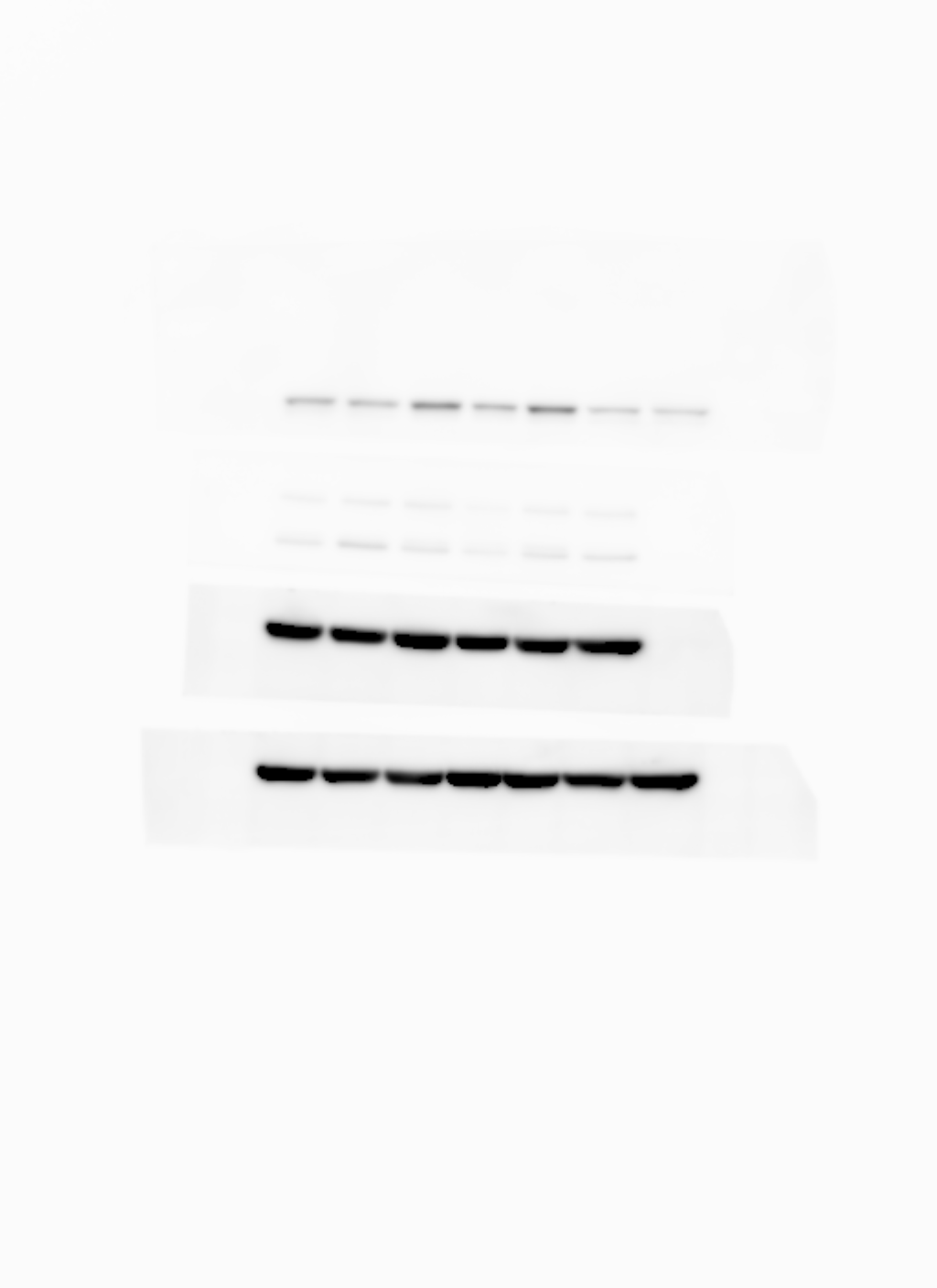


**NFκB**

**SOCS3**

**β-actin**

**25**

**30**

**Original images of immunoblots**

**Fig. 2a**

**(Kd)**


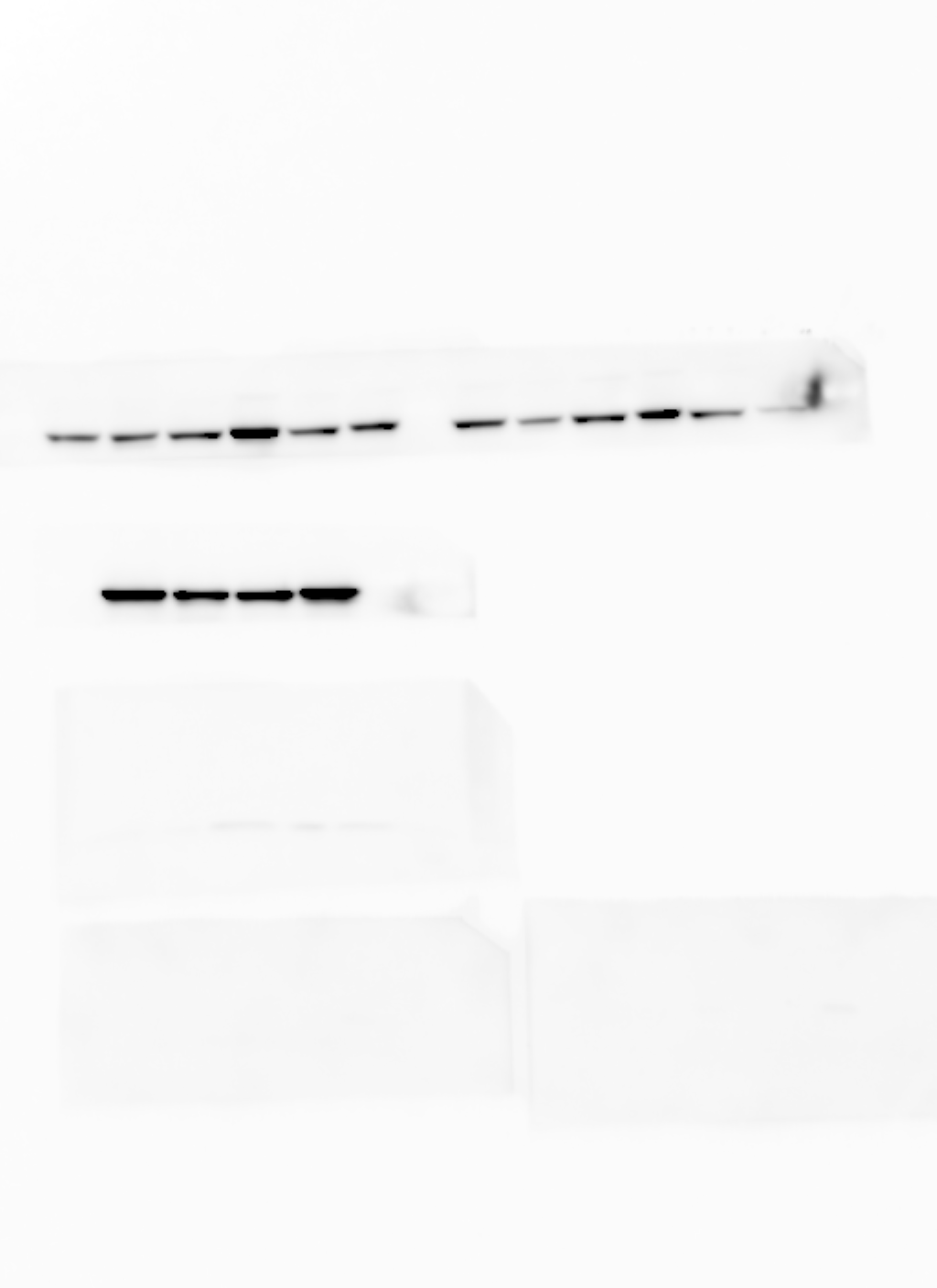


**70**


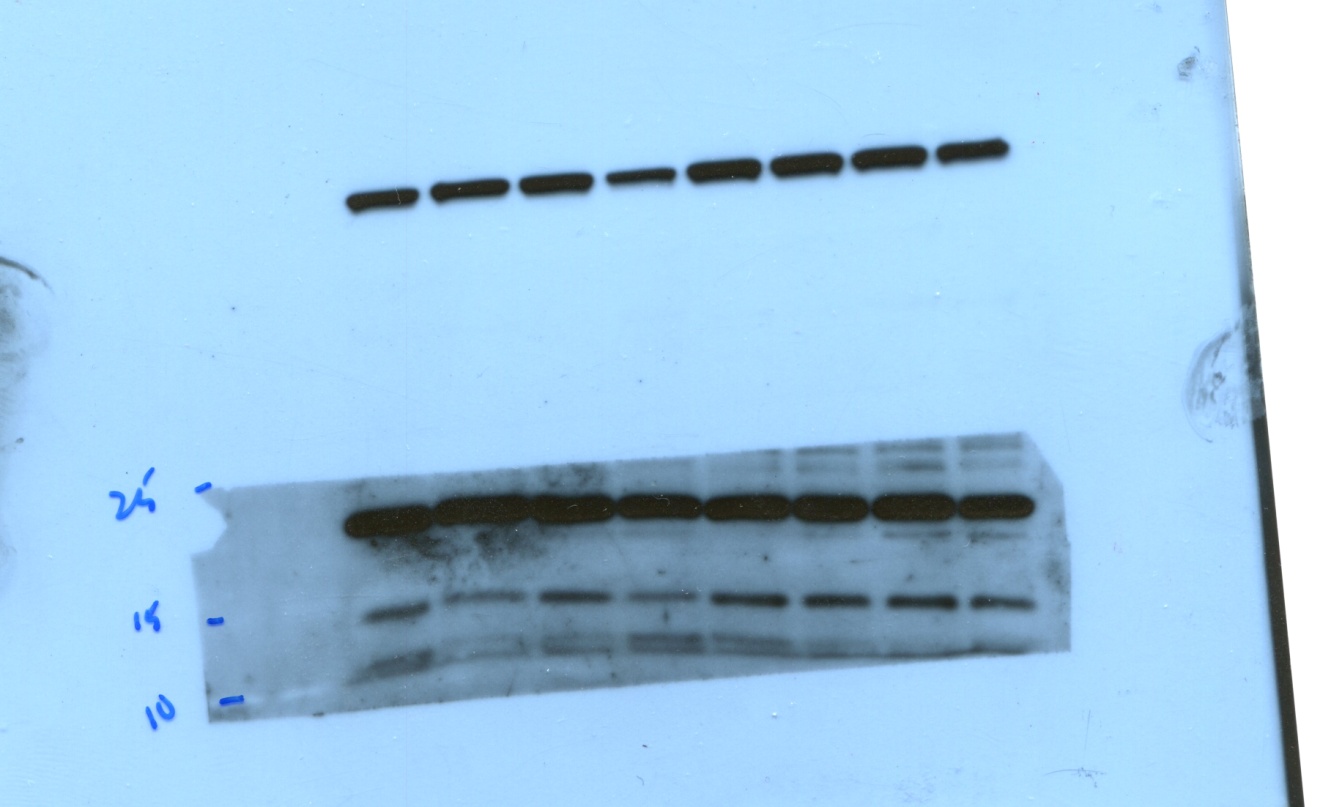


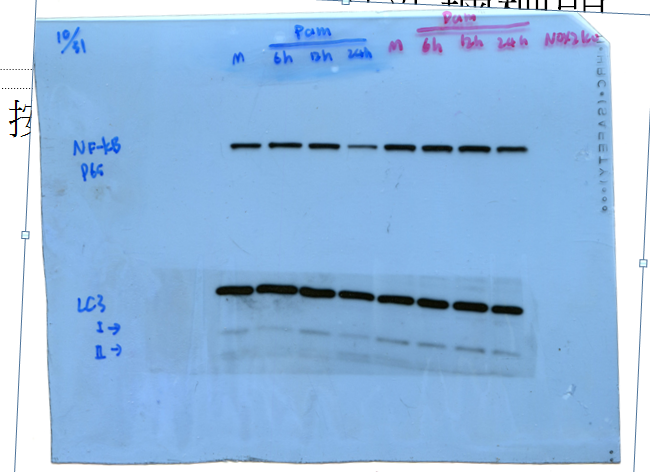


**NFκB**

**LC3I**

**LC3II**


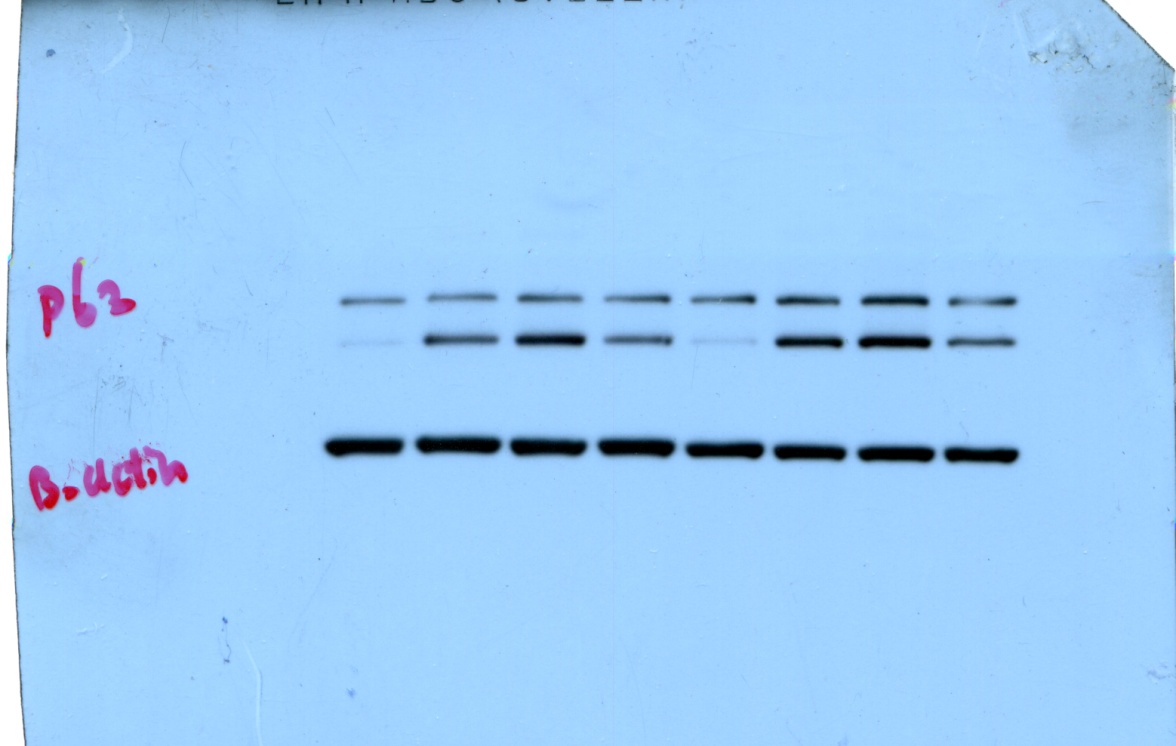


**(Kd)**

**70**

**15**

**10**

**Original images of immunoblots**

**Fig. 3e**


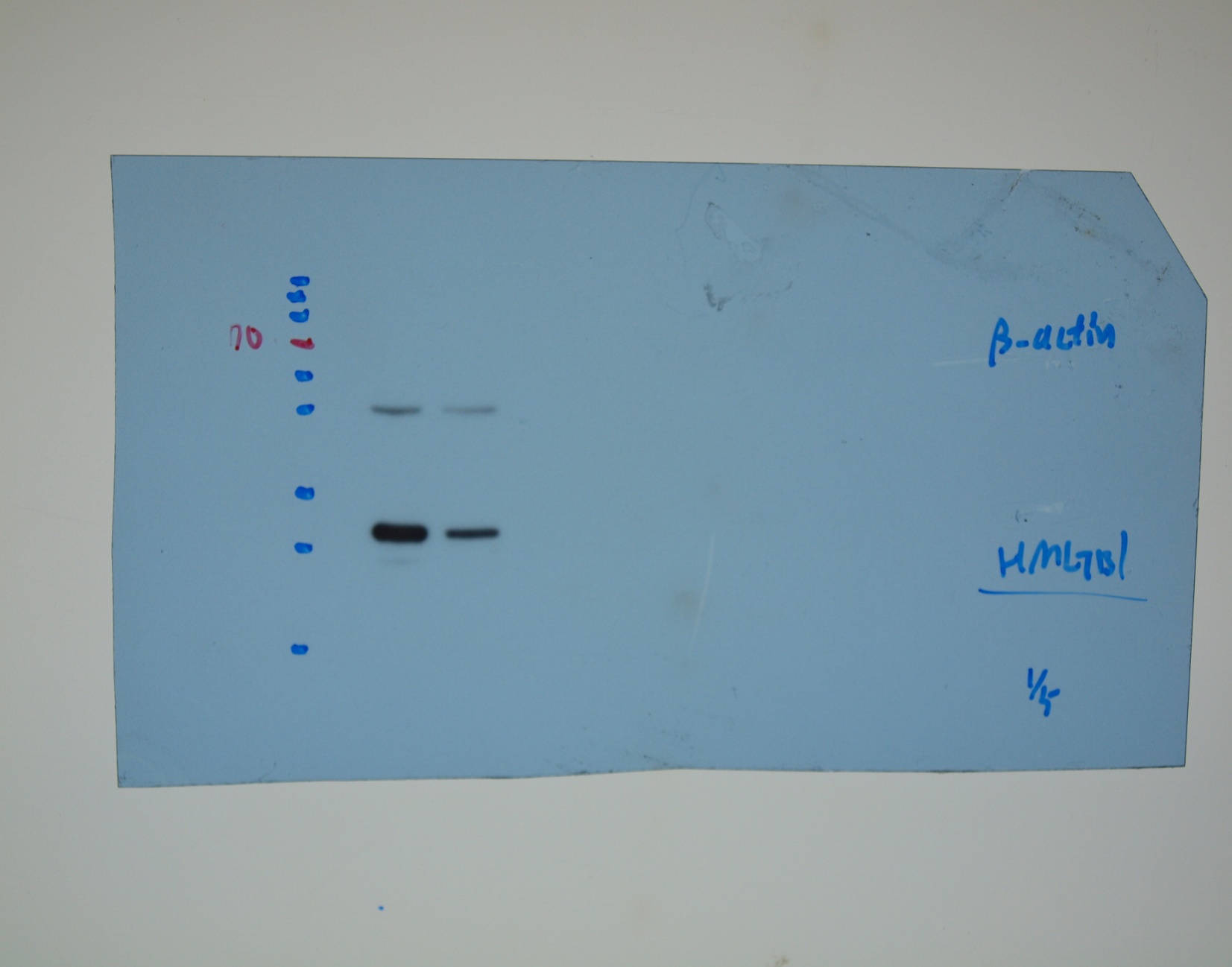

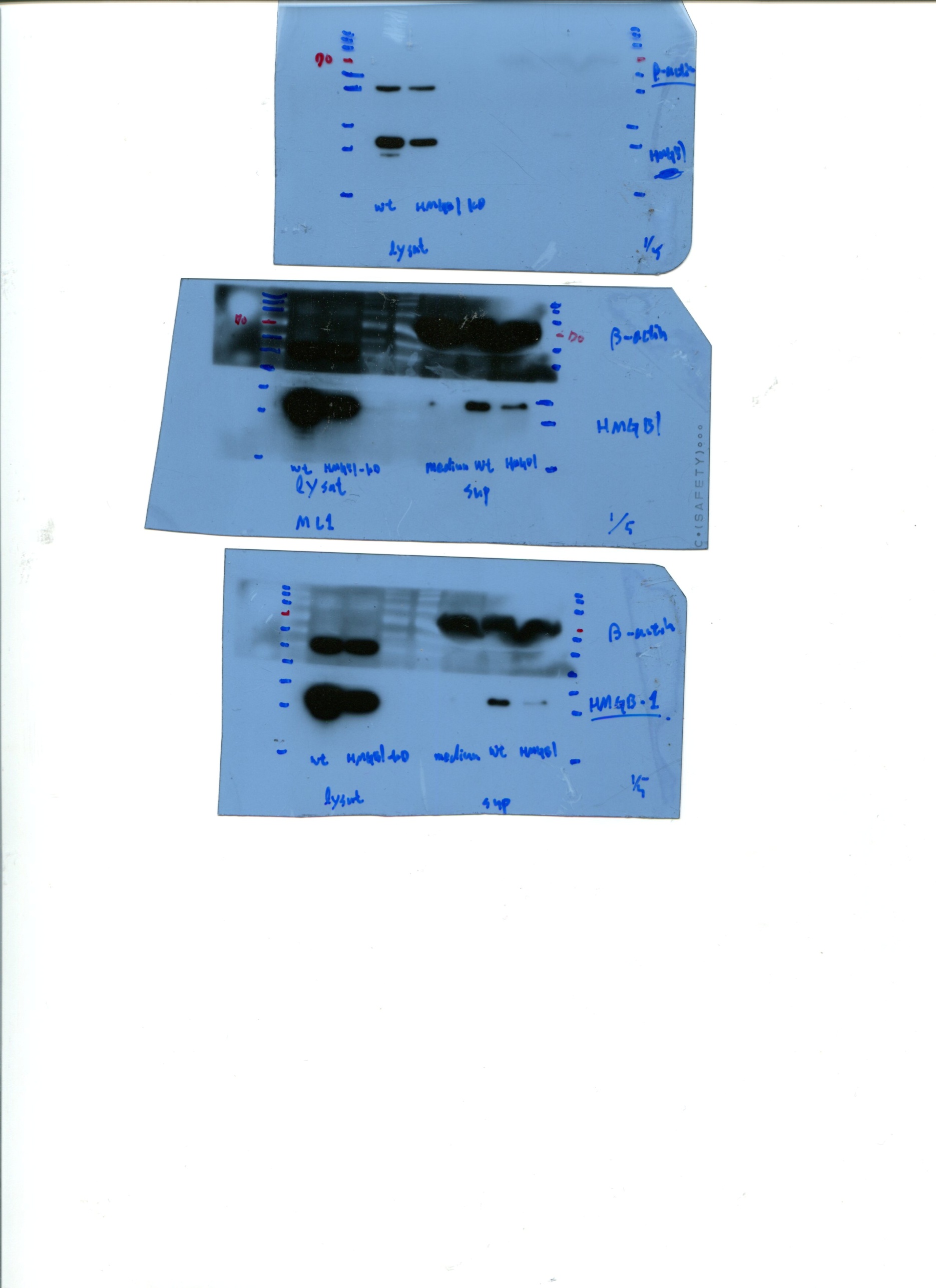

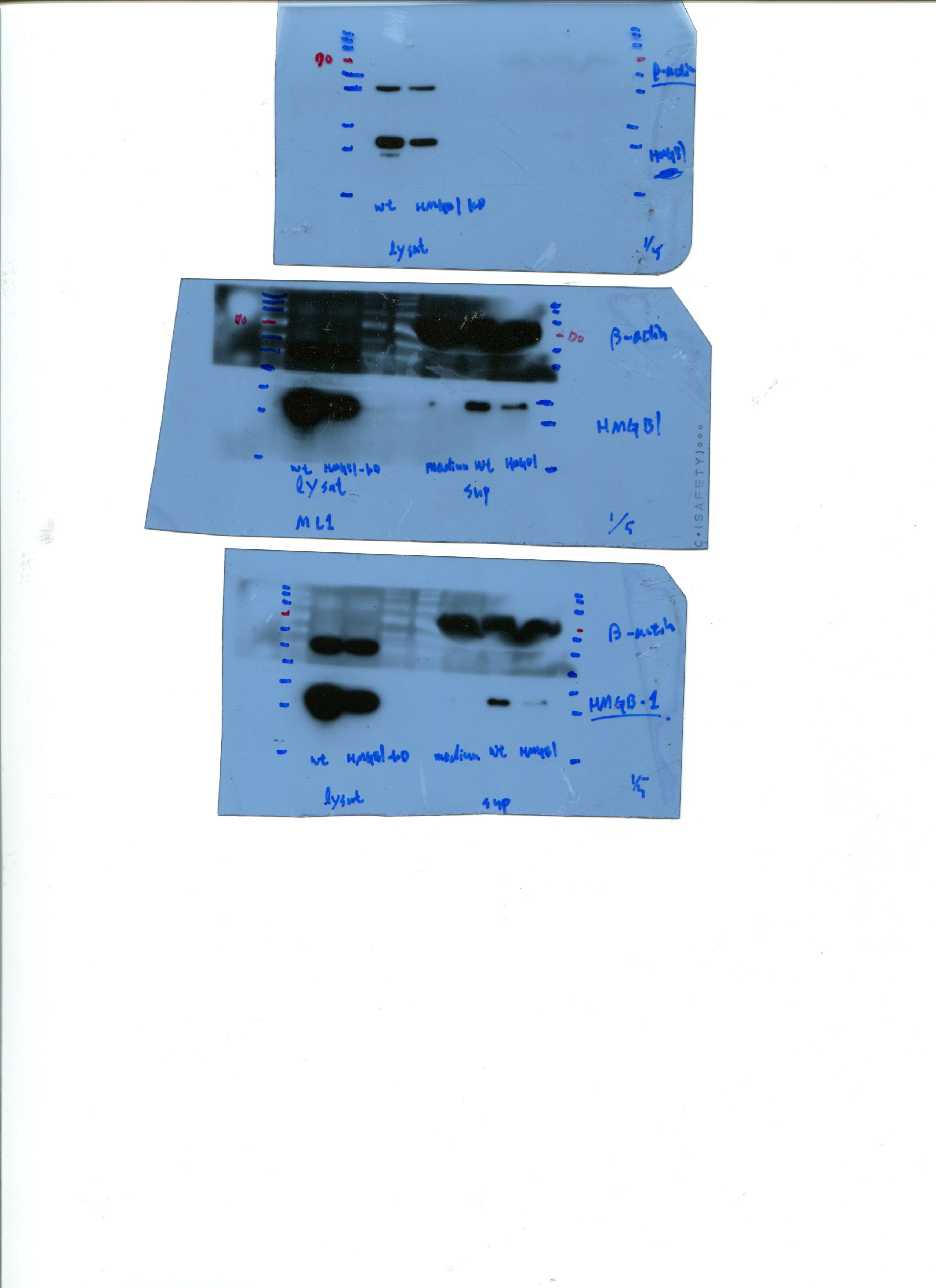

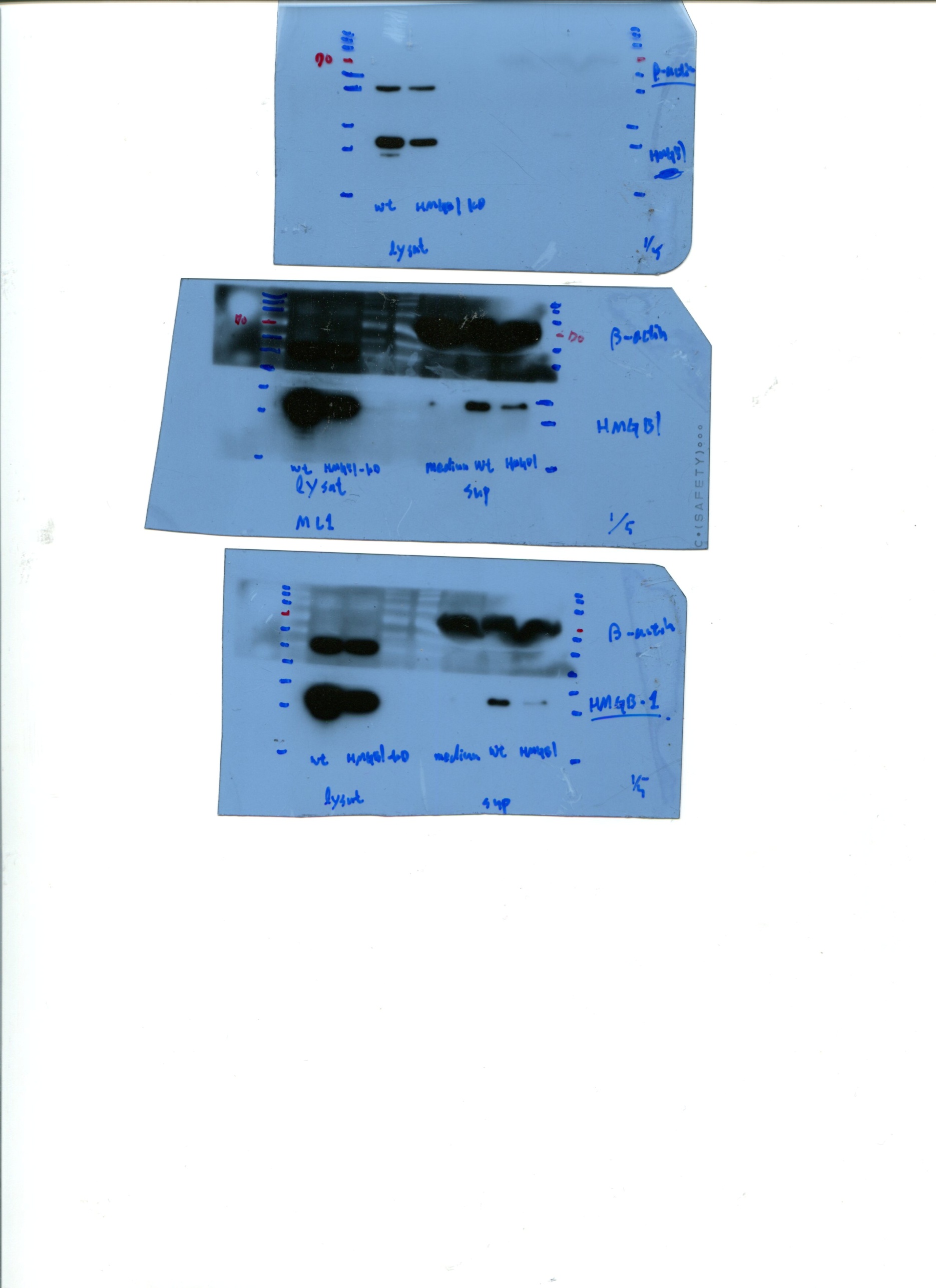


**HMGB1**

**β-actin**

**(Kd)**

**15**

**(Kd)**

**25**

**45**

**shLUC**

**shHMGB1**

**shLUC**

**shHMGB1**

**Cell lysate**

**Condition media**

**Original images of immunoblots**

**Fig. 4a**


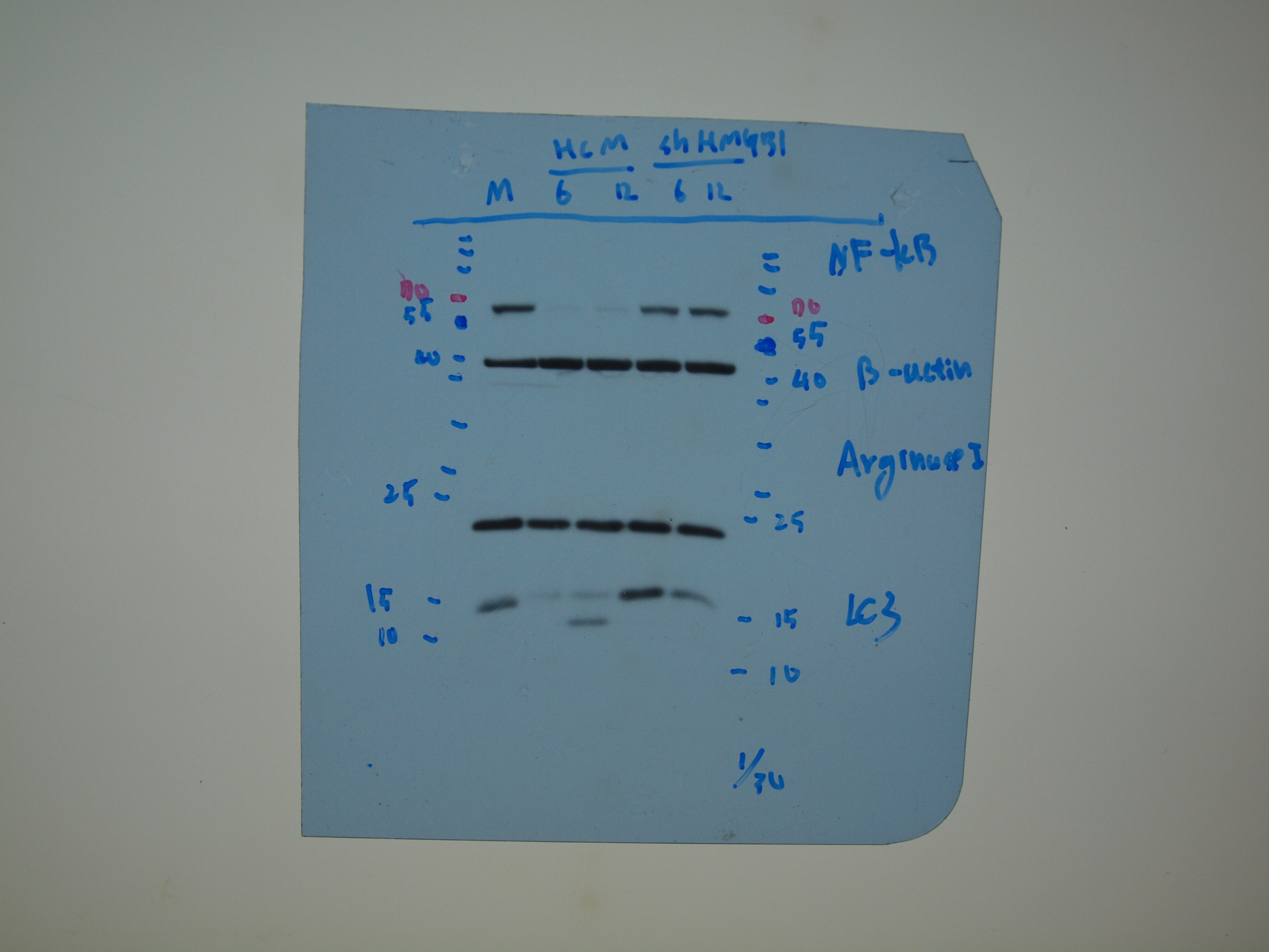

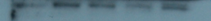

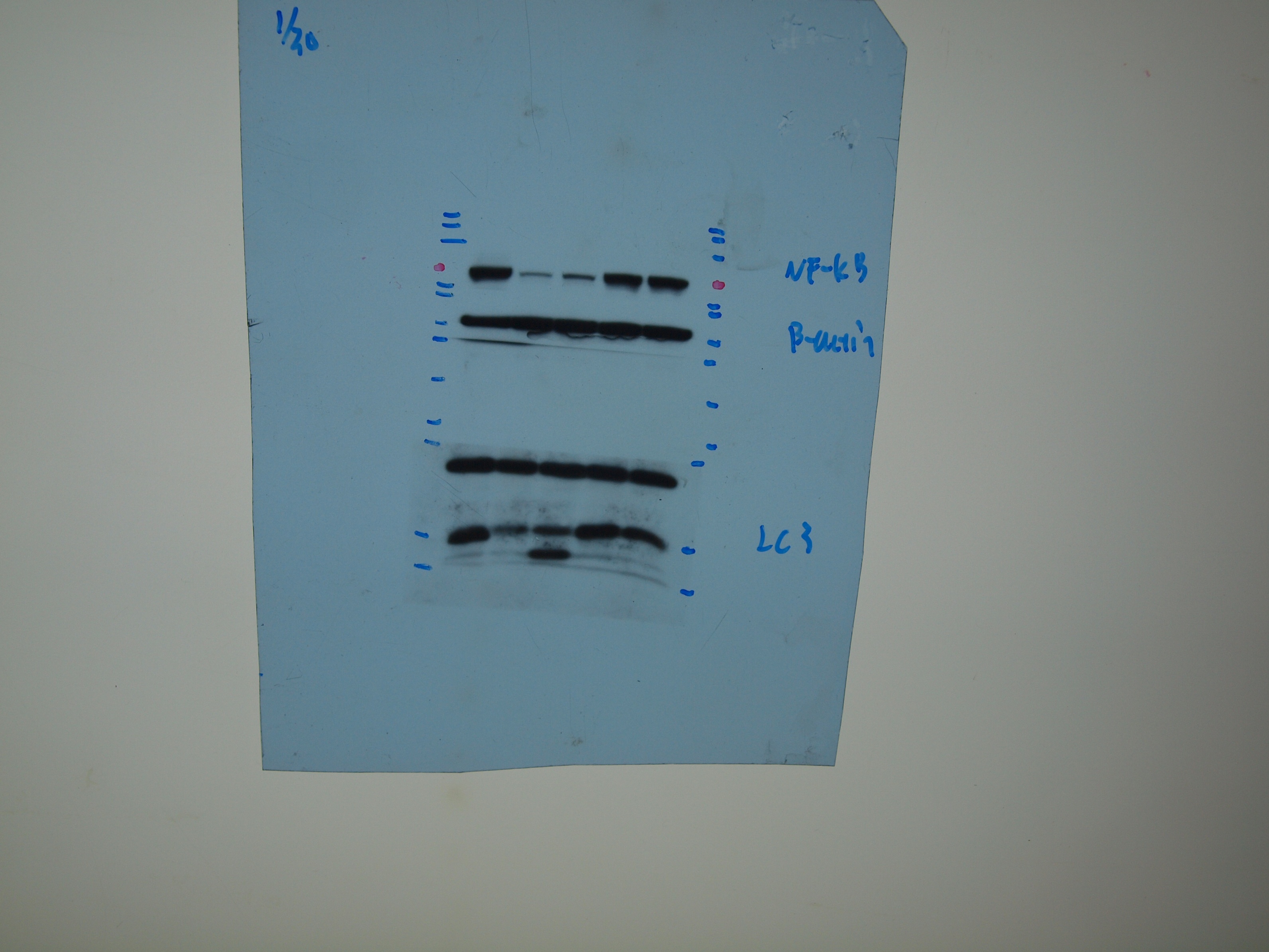


**Original images of immunoblots**

**Fig. 4d**

**Arginase 1 (long exposure)**


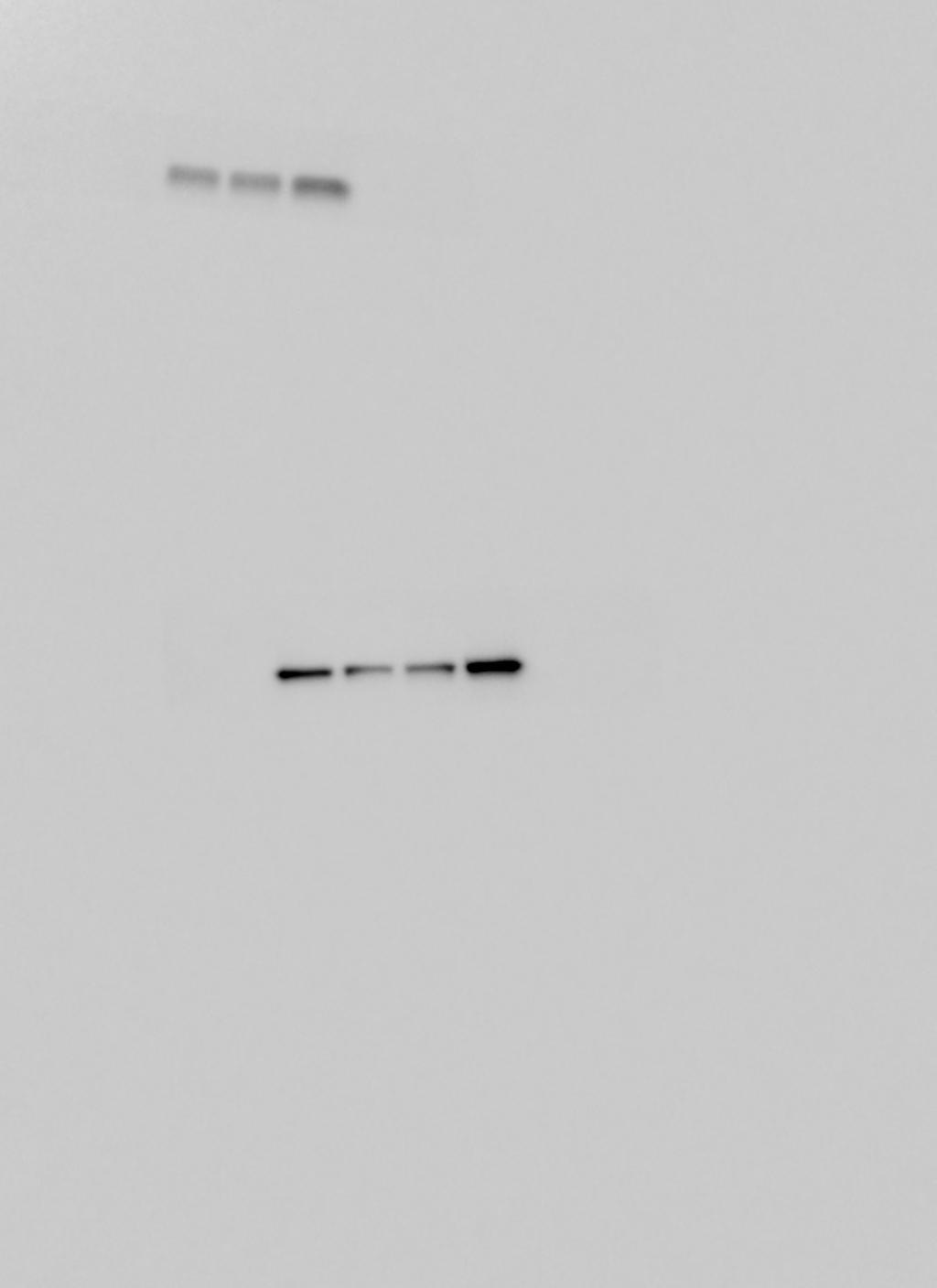

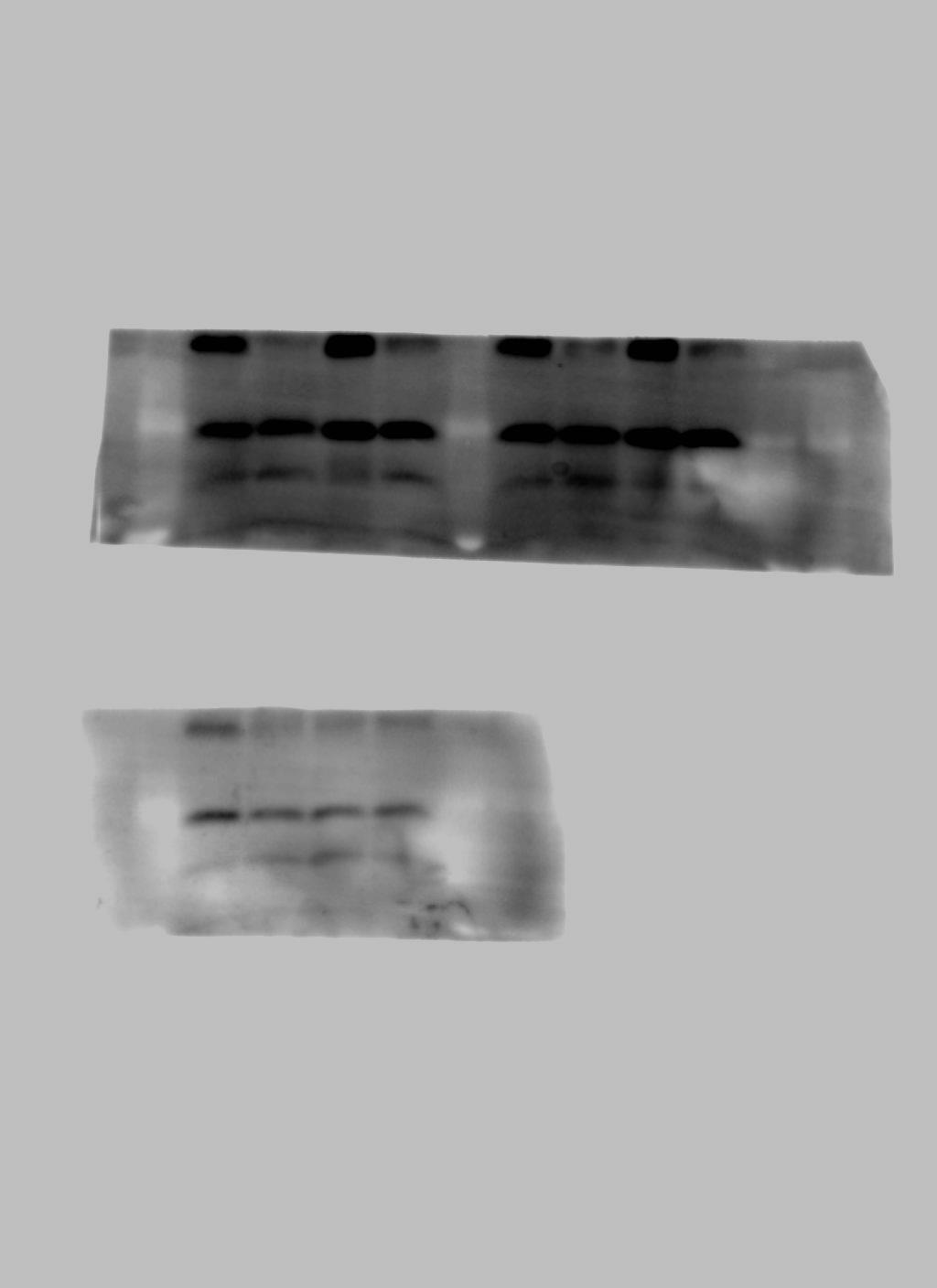

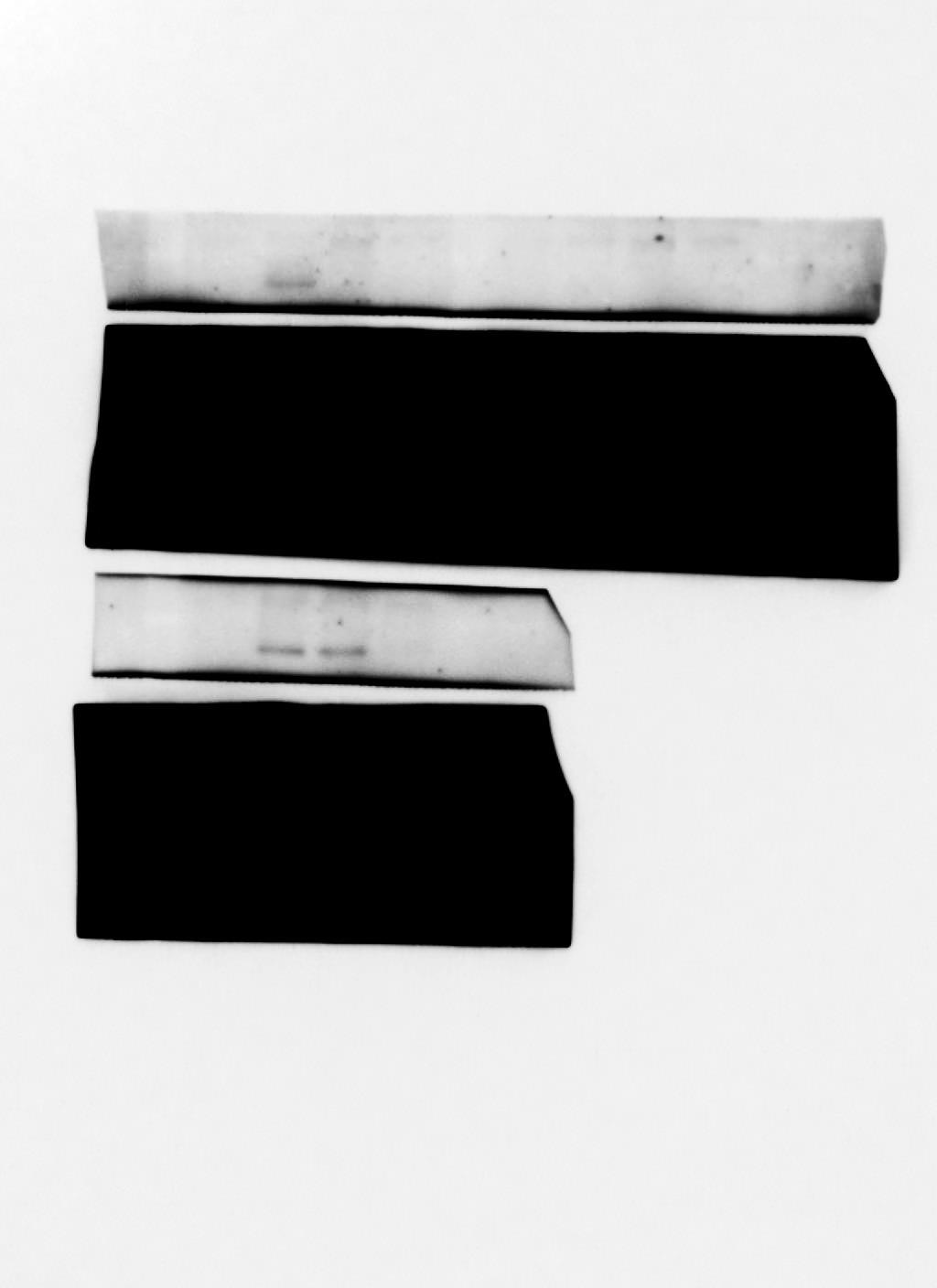

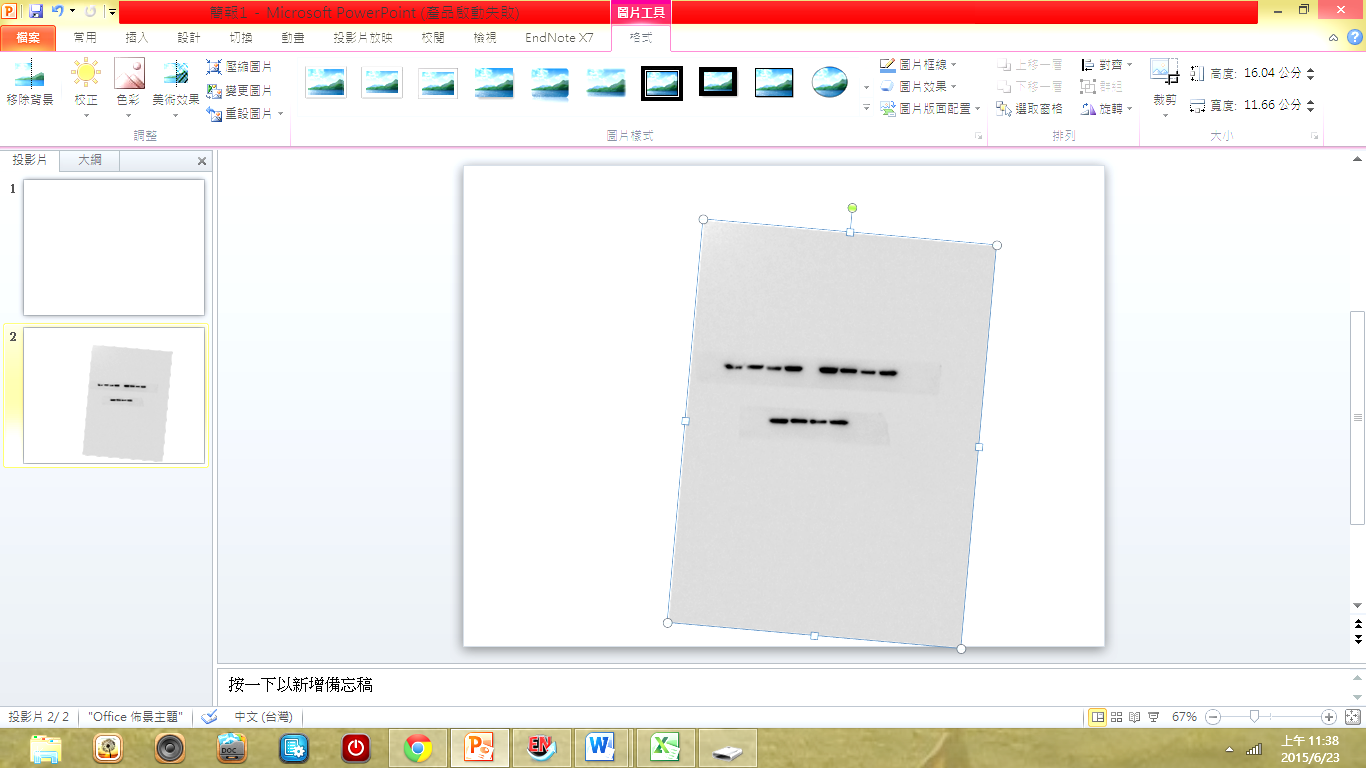


**NFκB**

**LC3I**

**LC3II**

**β-actin**

**Arginase I**

**70**

**15**

**10**

**55**

**55**

**Original images of immunoblots**

**Fig. 4g**


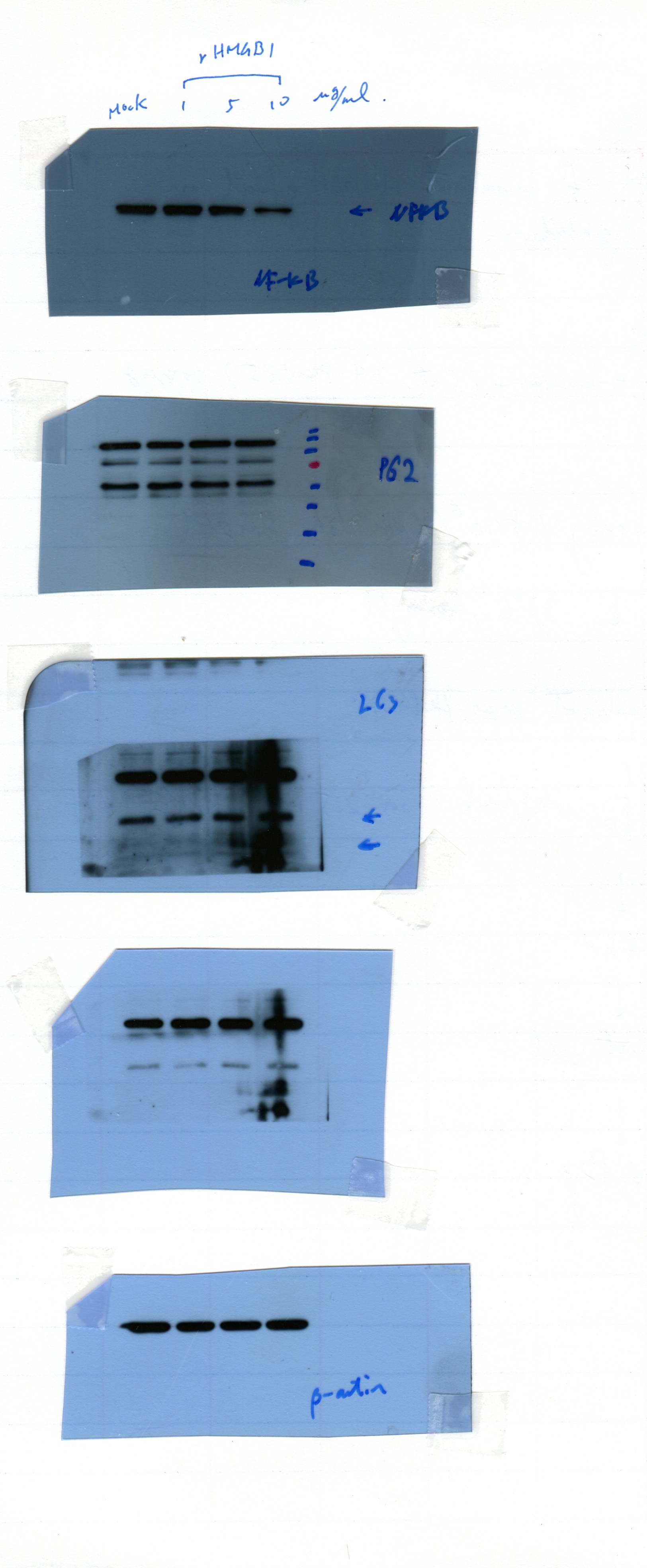

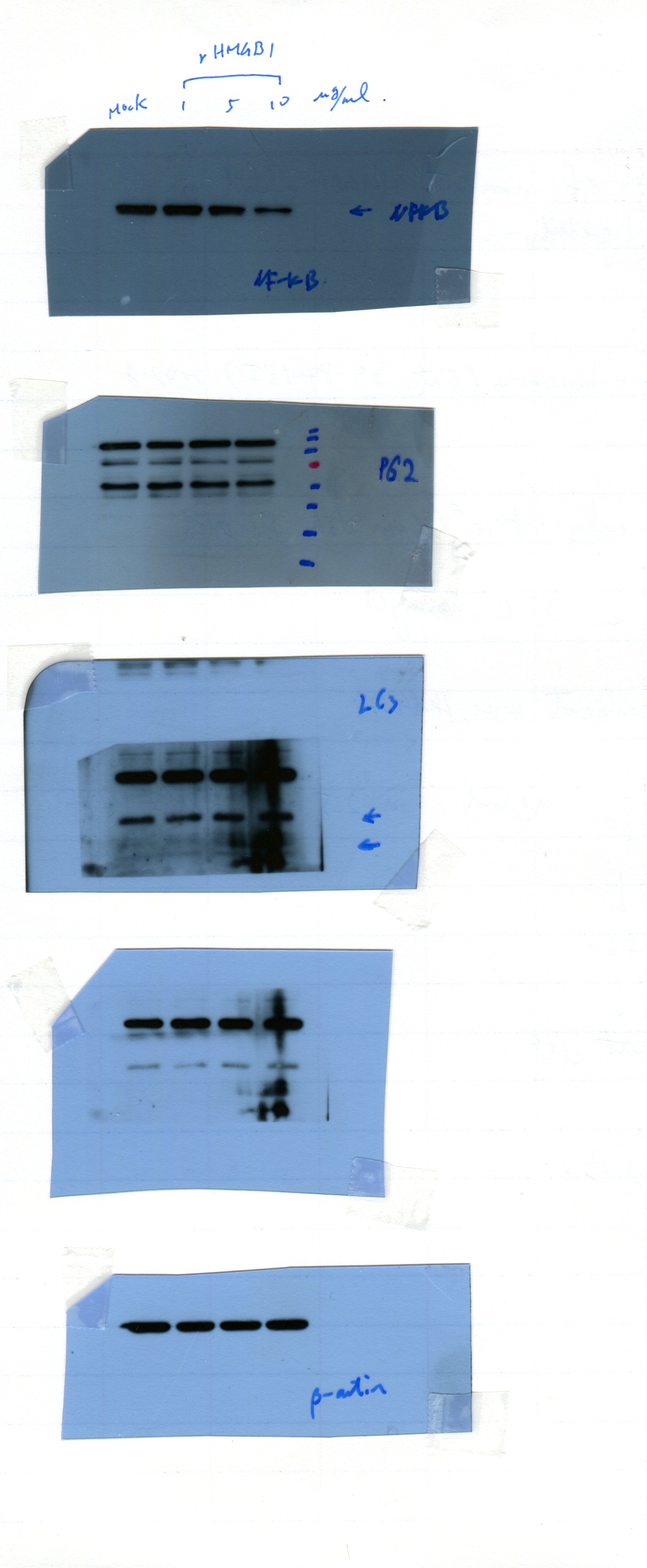

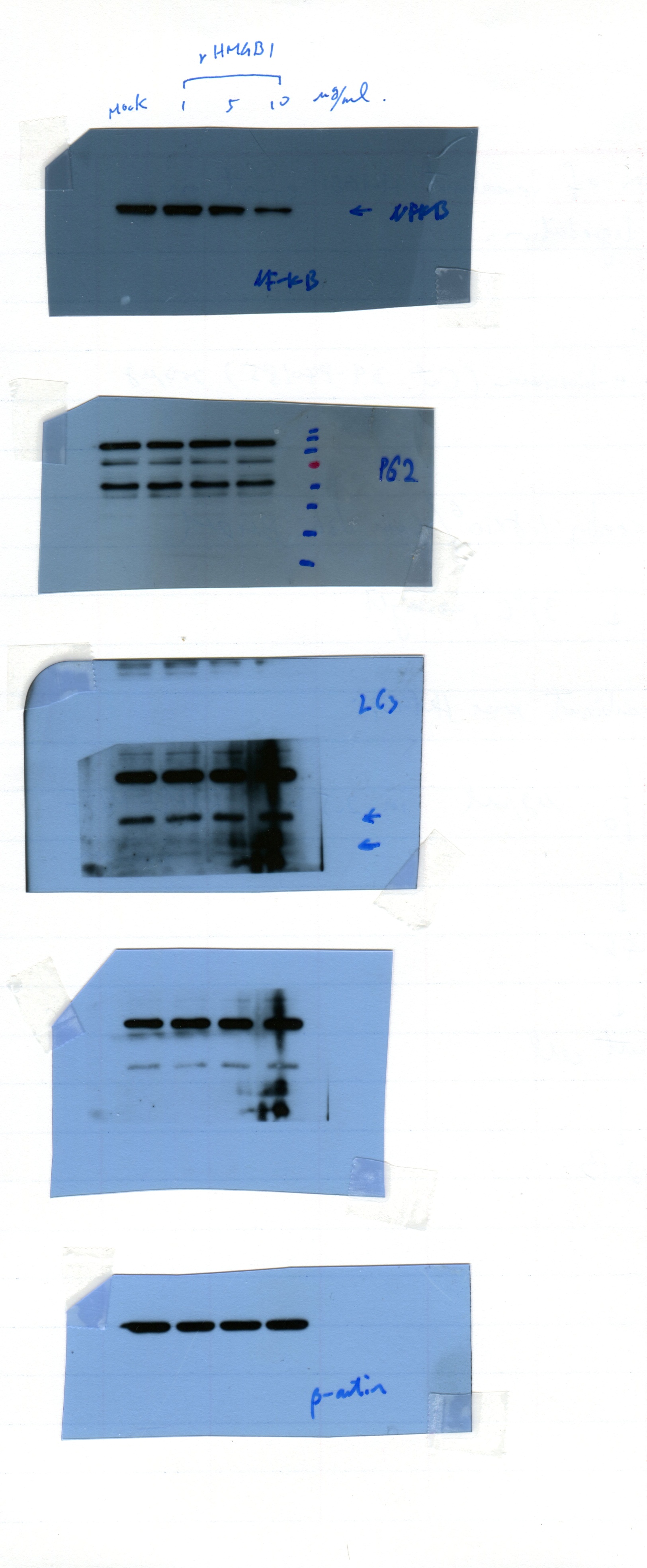


**NF-κB**

**LC3 I**

**β-actin**

**LC3 II**

**(Kd)**

**70**

**15**

**10**

**55**

**Original images of immunoblots**

**Fig. 5a**


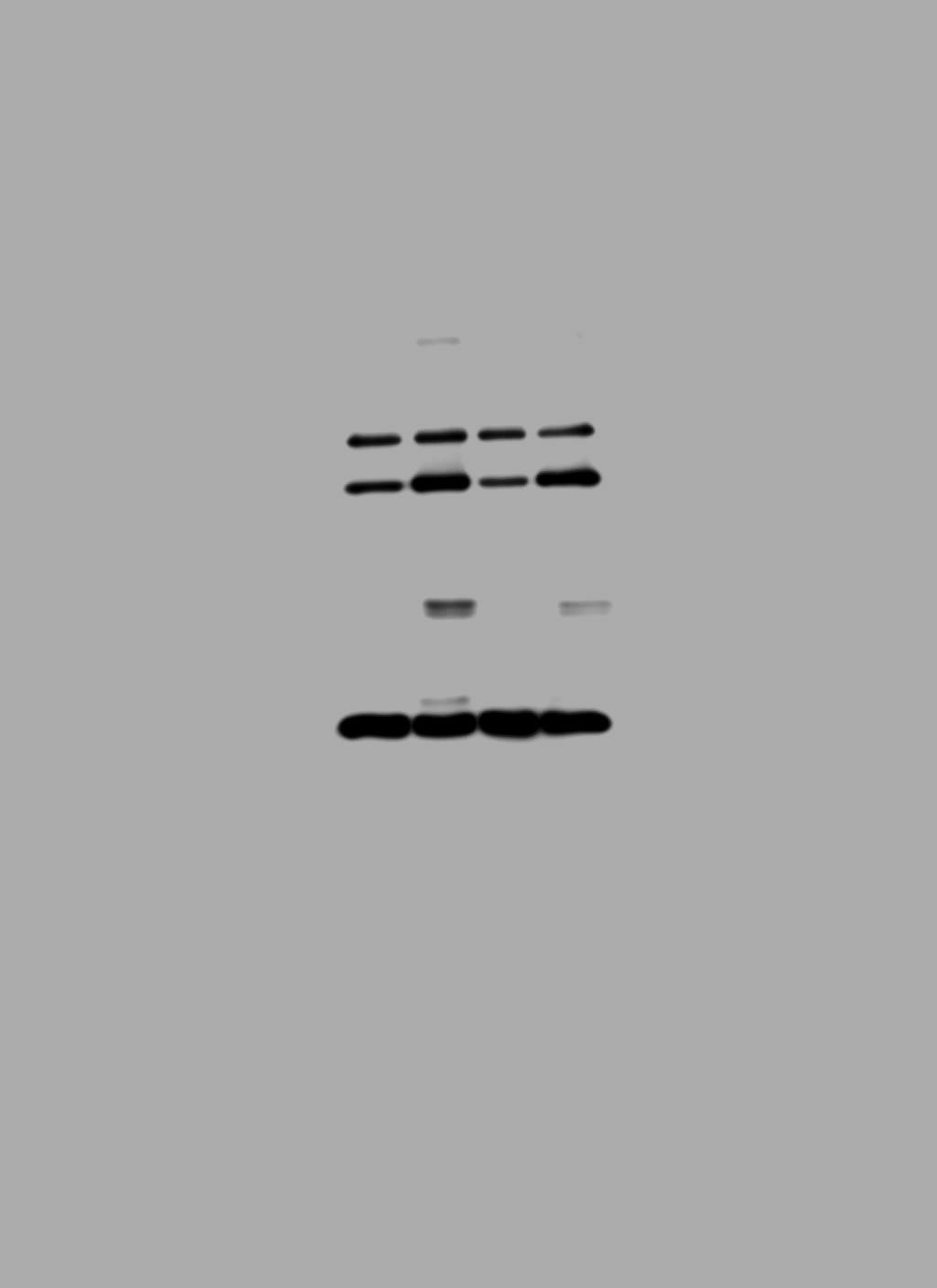

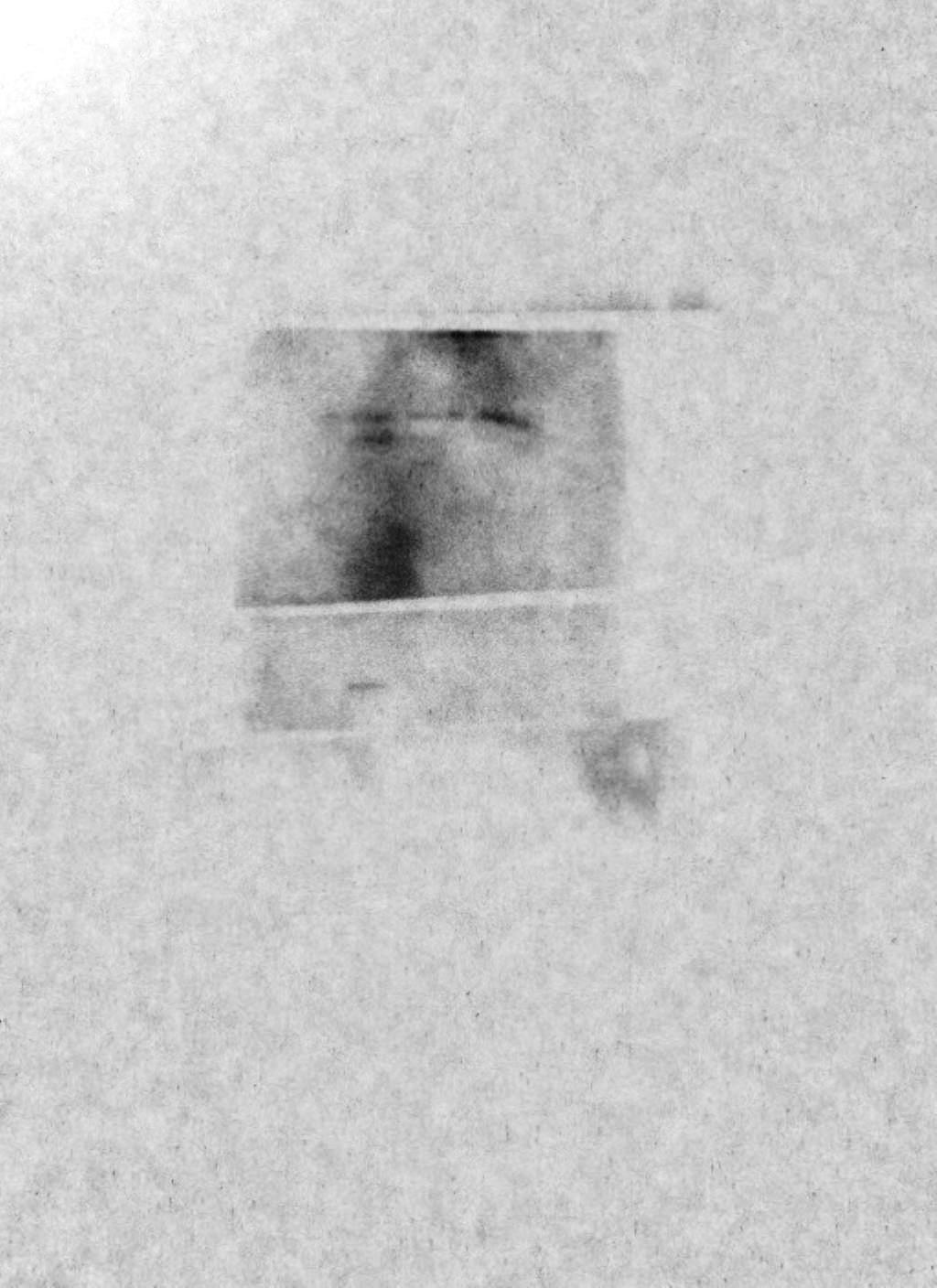

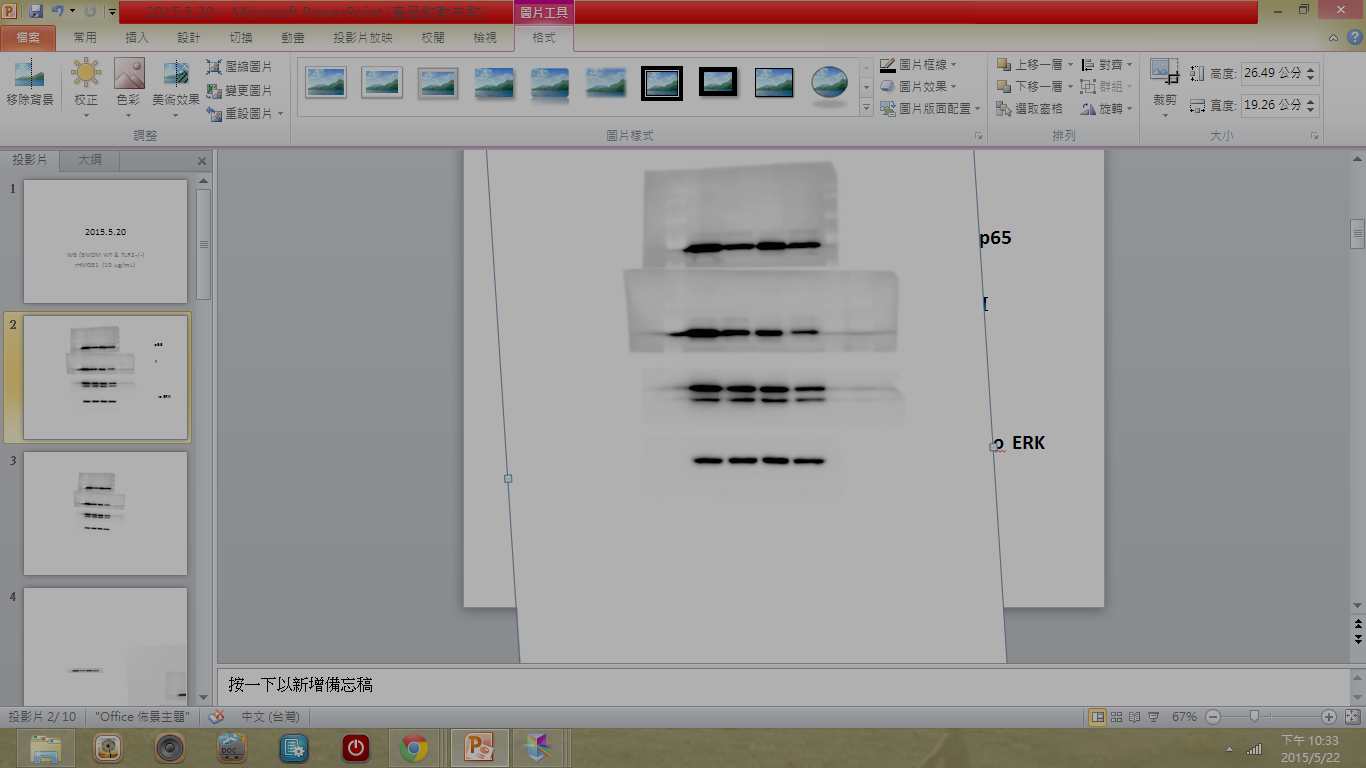


**β-actin**

**Arginase I**

**SOCS3**

**(Kd)**

**25**

**55**

**55**

**Original images of immunoblots**

**Fig. 5d**


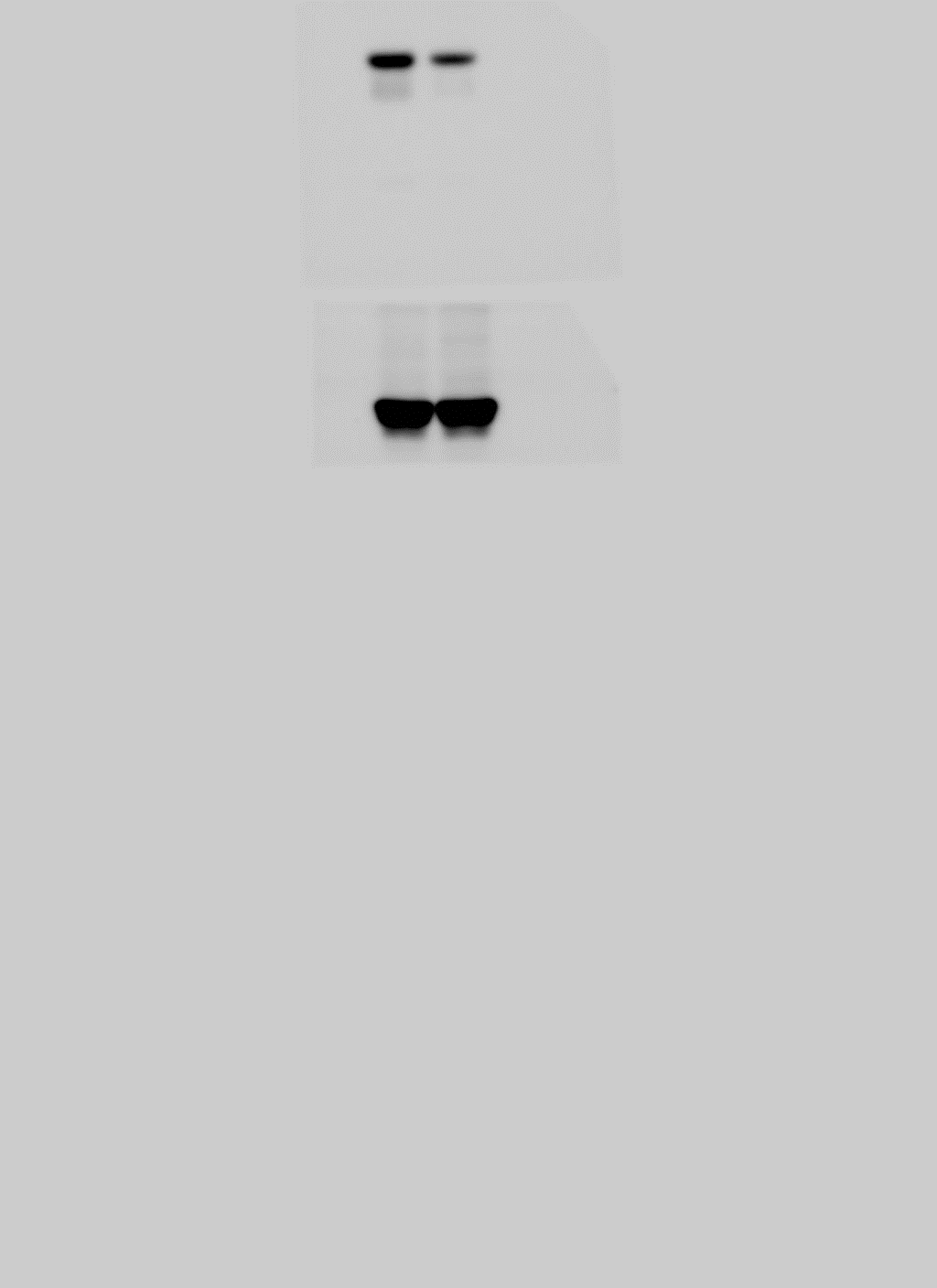

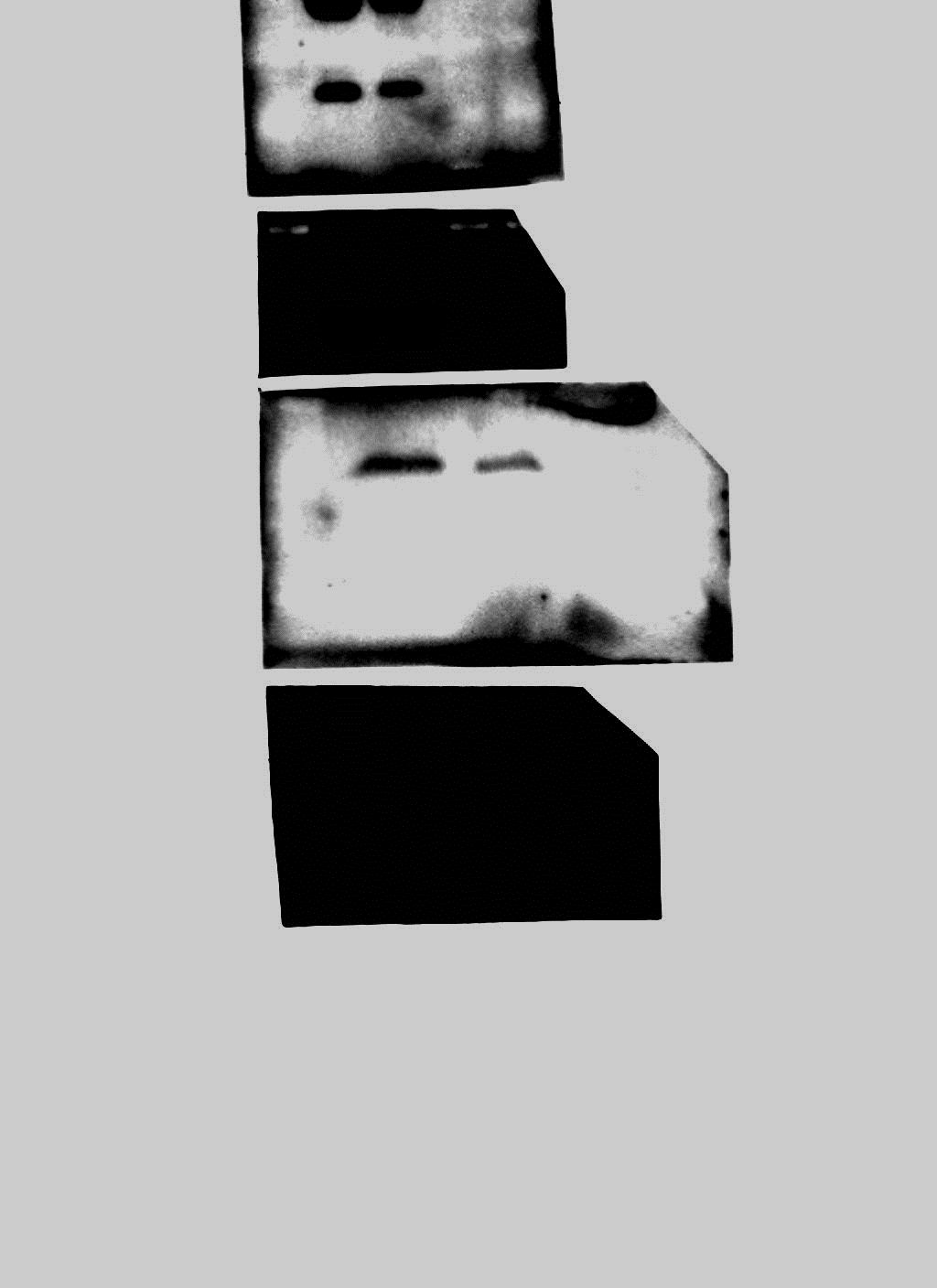

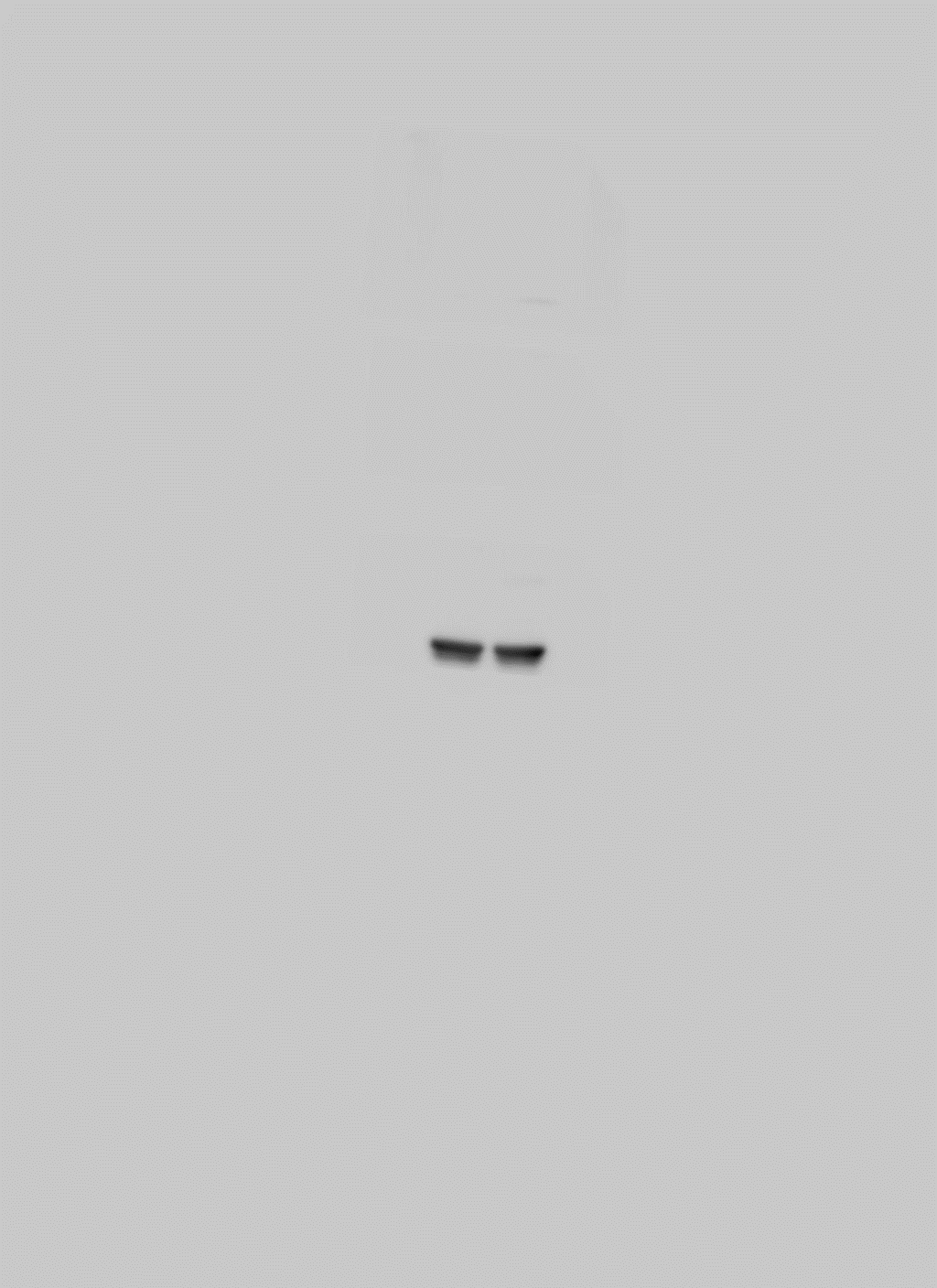


**Original images of immunoblots**

**Supplementary Fig. S1a**

**ML-1_4a_**

**MEF**

**Cytosolic HMGB1**

**Supernatant HMGB1**

**β-actin**

**25**

**55**

**25**
